# Genome-wide identification and characterization of small auxin-up RNA (*SAUR*) gene family in plants: evolution and expression profiles during normal growth and stress response

**DOI:** 10.1186/s12870-020-02781-x

**Published:** 2021-01-06

**Authors:** Hao Zhang, Zhenjia Yu, Xiaodie Yao, Jingli Chen, Xing Chen, Huiwen Zhou, Yuxia Lou, Feng Ming, Yue Jin

**Affiliations:** Shanghai Key Laboratory of Plant Molecular Sciences, College of Life Sciences, Shanghai, 200234 China

**Keywords:** SAUR, Auxin, Phylogenetic tree, Expression pattern

## Abstract

**Background:**

Auxin is critical to plant growth and development, as well as stress responses. *Small auxin-up RNA* (*SAUR*) is the largest family of early auxin responsive genes in higher plants. However, the function of few *SAUR* genes is known owing to functional redundancy among the many family members.

**Results:**

In this study, we conducted a phylogenetic analysis using protein sequences of 795 SAURs from *Anthoceros angustus*, *Marchantia polymorpha*, *Physcomitrella patens*, *Selaginella moellendorffii*, *Ginkgo biloba*, *Gnetum montanum*, *Amborella trichopoda*, *Arabidopsis thaliana*, *Oryza sativa*, *Zea mays*, *Glycine max*, *Medicago truncatula* and *Setaria italica*. The phylogenetic trees showed that the SAUR proteins could be divided into 10 clades and three subfamilies, and that SAUR proteins of three bryophyte species were only located in subfamily III, which suggested that they may be ancestral. From bryophyta to anthophyta, *SAUR* family have appeared very large expansion. The number of *SAUR* gene in Fabaceae species was considerably higher than that in other plants, which may be associated with independent whole genome duplication event in the Fabaceae lineages. The phylogenetic trees also showed that *SAUR* genes had expanded independently monocotyledons and dicotyledons in angiosperms. Conserved motif and protein structure prediction revealed that SAUR proteins were highly conserved among higher plants, and two leucine residues in motif I were observed in almost all SAUR proteins, which suggests the residues plays a critical role in the stability and function of SAUR proteins. Expression analysis of *SAUR* genes using publicly available RNA-seq data from rice and soybean indicated functional similarity of members in the same clade, which was also further confirmed by qRT-PCR. Summarization of SAUR functions also showed that SAUR functions were usually consistent within a subclade.

**Conclusions:**

This study provides insights into the evolution and function of the *SAUR* gene family from bryophyta to anthophyta, particularly in Fabaceae plants. Future investigation to understand the functions of *SAUR* family members should employ a clade as the study unit.

**Supplementary Information:**

The online version contains supplementary material available at 10.1186/s12870-020-02781-x.

## Background

As sessile organisms, plants must adapt to environmental variation by integrating developmental and environmental signals. Auxin was the first plant hormone to be identified, and plays important roles in plant growth, development and stress responses [1]. Three gene families are regarded as early and primary auxin-responsive genes: *Auxin/Indoleacetic Acid* (*Aux/IAA*), *Gretchen Hagen 3* (*GH3*) and *Small Auxin-Up RNA* (*SAUR*) [2, 3]. *SAUR* genes can be induced originally by exogenous auxin within 2–5 min [4]. Interestingly, the majority of *SAUR* genes lack an intron [3, 5], and may be regulated at a number of levels; for example, *SAUR* genes can be regulated post-transcriptionally owing to a highly conserved downstream element (DST) located in the 3′-untranslated region, which leads to mRNA instability in an auxin-independent manner [1, 6–8].

The first *SAUR* gene was identified from elongating hypocotyls of auxin-treated soybean (*Glycine max*) [9]. Over the past 30 years, members of the *SAUR* gene family have been identified in many other species of angiosperms and thus comprise a large gene family in plants, such as *Arabidopsis thaliana* (81 *SAUR* genes, including two pseudogenes) [2], *Oryza sativa* (58 including two pseudogenes) [10], *Zea mays* (79) [3], *Gossypium raimondii* (145) [11], *Citrus sinensis* (70) [12], *Phyllostachys edulis* (44) [13], *Boehmeria nivea* (71) [14] and *Citrullus lanatus* (65) [15]. Many members of the *SAUR* gene family are a result of a high frequency of tandem and segmental duplications, and which have contributed to functional redundancy among the paralogues [6].

Until now, several *SAUR* genes have been shown to play roles in diverse processes of plant growth, development and stress responses. For example, *AtSAUR63* promotes cell elongation, thus resulting in longer hypocotyls, stamen filaments, petals and inflorescence stems [16]. Overexpression of *AtSAUR36* and *AtSAUR49* promotes leaf senescence [17–19]. The *atsaur62* and *atsaur75* mutants show normal pollen viability but defective pollen tube growth in vitro and in vivo [20]. The SAUR50-like protein is involved in heliotropic movements in the common sunflower (*Helianthus annuus)* [21]. Many *SAUR* homologues play crucial roles in circadian floral opening and closure in waterlily [22, 23]. The *SAUR41* subfamily is inducible by abscisic acid to modulate cell expansion and salt tolerance in *Arabidopsis thaliana* seedlings [24]. The thermo-responsive *AtSAUR26* subfamily exhibits a high frequency of gene variation associated with adaption to local temperature climate [25].

To improve our understanding of the evolution and functions of the *SAUR* gene family in plants, in this study we first conducted a phylogenetic analysis using 795 SAUR protein sequences from *Anthoceros angustus*, *Marchantia polymorpha*, *Physcomitrella patens*, *Selaginella moellendorffii*, *Ginkgo biloba*, *Gnetum montanum*, *Amborella trichopoda*, *Arabidopsis thaliana*, *Oryza sativa*, *Zea mays*, *Setaria italica*, *Glycine max* and *Medicago truncatula*. Conserved motif and protein structure prediction and gene expression profiling of the *SAUR* family were performed to explore possible functions of *SAUR* genes. In addition, we summarized functions of all identified *SAUR* genes.

## Results

### SAUR protein identification from alga to higher plant species

We identified the SAUR protein family members from the following plant species in BLAST searches of the Phytozome 12 database: *Physcomitrella patens* (18 members), *Arabidopsis thaliana* (81, including two pseudogenes), *Oryza sativa* (58, including two pseudogenes), and maize (79). This was consistent with the results of previous studies [2, 3, 10, 26]. Meanwhile, we firstly identified 3, 5 and 15 SAUR protein sequences in *Anthoceros angustus*, *Marchantia polymorpha* and *Selaginella moellendorffii*, respectively. In gymnosperm, *SAUR* family members of *Ginkgo biloba* (42) and *Gnetum montanum* (37) were firstly identified. For anthophyta, 26 and 58 SAUR protein sequences were firstly identified in *Amborella trichopoda* and *Setaria italica*, respectively. Interestingly, up to 141 and 236 SAUR protein sequences were identified in *Medicago truncatula* and soybean. These results indicated that *SAUR* family members had expanded largely in the process of evolution. In addition, none *SAUR* homolog was found in genomes of the seven chlorophyte species (*Chlamydomonas reinhardtii*, *Dunaliella salina*, *Volvox carteri*, *Coccomyxa subellipsoidea* C-169, *Micromonas pusilla* CCMP1545, *Micromonas* sp. RCC299 and *Ostreococcus lucimarinus*) and four recently annotated streptophyte algae species (*Spirogloea muscicola*, *Mesotaenium endlicherianum*, *Mesostigma viride* and *Chlorokybus atmophyticus*) using SAUR protein sequences of *Arabidopsis thaliana* as queries. All identified SAUR protein sequences from different species were used for downstream analysis (Supplementary Table 1).

The encoded SAUR proteins were polypeptides of 55–423 amino acids in length, with a predicted molecular mass range of 6.38–46.81 kDa and the theoretical isoelectric point (pI) ranged from 4.58 to 12. In further analysis, we used the Plant-mPLoc server (http://www.csbio.sjtu.edu.cn/bioinf/plant-multi/) [27] to predict the probable protein localization of identified SAURs. More than 60.8% of the SAUR proteins contained a nucleus-targeting signal and the other proteins were predicted to be localized to the cell membrane, cytoplasm, chloroplasts or mitochondria, etc. (Supplementary Table 2).

### Phylogenetic analysis of SAUR proteins

**Fig. 1:**
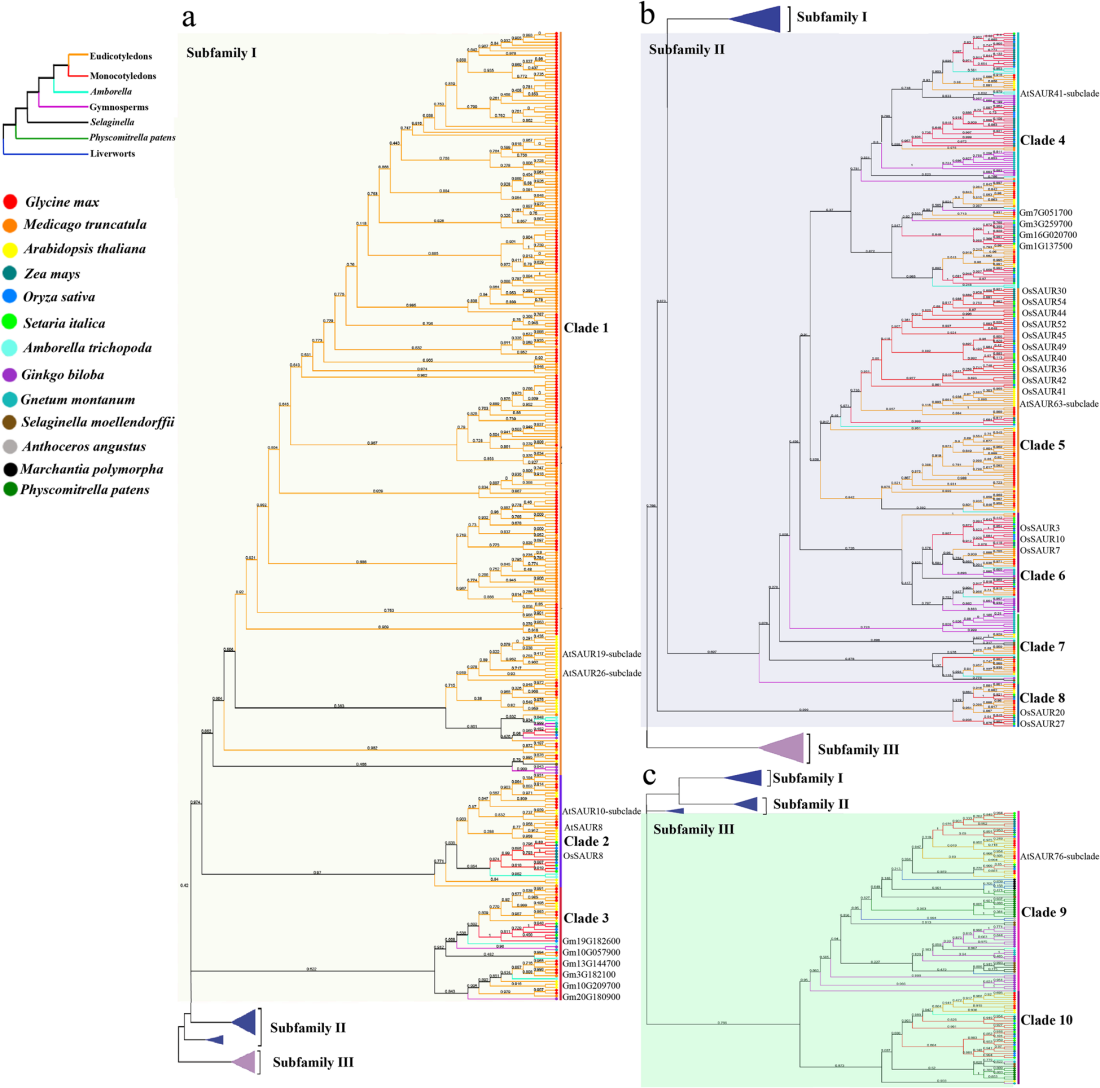
Maximum likelihood phylogenetic tree of small auxin-up RNAs (*SAURs*) in the thirteen plant species. The tree was constructed from multiple sequences alignment of all SAUR proteins from *Anthoceros angustus*, *Marchantia polymorpha*, *Physcomitrella patens*, *Selaginella moellendorffii*, *Ginkgo biloba*, *Gnetum montanum*, *Amborella trichopoda*, *Arabidopsis thaliana*, *Oryza sativa*, *Zea mays*, *Setaria italica*, *Glycine max* and *Medicago truncatula* using the maximum likelihood method in FastTree v2.1 with the JTT + CAT model. Then SAUR members were clarified in FigTree v1.4.4. There SAUR family had been divided into three subfamily and 10 clades. Bootstrap support rates were labeled at corresponding branches. SAUR subclades reported and members mentioned in the article were also labeled on it. Branches leading to genes from the different phyla were colored according to the simplified phylogeny of land plants that is shown in the top left corner. Different colorful dots were used to represent SAUR members from different species

To gain insights into the evolution of *SAUR* genes in plants, we used SAUR protein sequences, excluding pseudogenes, from *Anthoceros angustus* (3), *Marchantia polymorpha* (5), *Physcomitrella patens* (18), *Selaginella moellendorffii* (15), *Ginkgo biloba* (42), *Gnetum montanum* (37), *Amborella trichopoda* (26), *Arabidopsis thaliana* (79), *Oryza sativa* (56), *Zea mays* (79), *Setaria italica* (58), *Glycine max* (236), and *Medicago truncatula* (141) to construct two phylogenetic trees for the SAUR protein family via FastTree v2.1 and IQ-TREE v2.0.6 respectively. Based on these two trees, we divided the SAUR proteins into three subfamilies and 10 clades designated groups 1 to 10, although there was a slight difference in several clades between the two trees (Fig. 1, Supplementary Fig. 1, Supplementary Datasheets 1, 2). All SAUR proteins of three bryophyte species were placed in two clades of subfamily III of the two trees, which suggested that these clades may be ancestral. The majority of monocotyledon and eudicotyledon sequences were each grouped together in one clade, which indicated that the *SAUR* gene family expanded independently in monocotyledons and eudicotyledons. Most SAUR members of *Ginkgo biloba*, *Gnetum montanum* had formed independent branches in parallel with ones of anthophyta, which was consistent with their evolutionary status. Most of members of clade 1 in both FastTree and IQ-TREE trees were proteins from soybean and *Medicago truncatula* (Fig. 1, Supplementary Fig. 1). This may be a result of gene duplication in the clade or independent genome replication among Fabaceae plants. In addition, both FastTree and IQ-TREE trees showed that the Fabaceae species formed a distinct evolutionary lineage (clade 1) and the number of SAUR protein members of eudicotyledons was roughly equal in their other shared branches (Fig. 1, Supplementary Fig. 1). We further reconstructed the phylogenetic tree using *SAUR* gene family members from *Mimosa pudica* and *Cercis canadensis*, which were sister taxa outside of Fabaceae family, together with *Arabidopsis thaliana*, *Glycine max, Medicago truncatula*. The phylogenetic tree could be divided ten clades, and *SAUR* gene family from *Medicago truncatula* and *Glycine max* only had extremely large expansion in Clade 1 (Supplementary Fig. 2). Synteny analysis is a useful tool for establishing both orthology relationships and functional linkages between genes. Then we performed synteny analysis of *SAUR* genes from *Arabidopsis thaliana*, *Glycine max* and *Medicago truncatula* (Supplementary Fig. 3). The results showed that *SAUR* gene cluster at a certain chromosome of *Arabidopsis thaliana* had evident synteny relationships with multiple *SAUR* gene clusters at multiple chromosomes of *Medicago truncatula* and *Glycine max*. These results suggested that the occurrence of many *SAUR* members in Fabaceae resulted from independent genome replication in Fabaceae.

### Conserved core region and structure specific to SAUR proteins

**Fig. 2:**
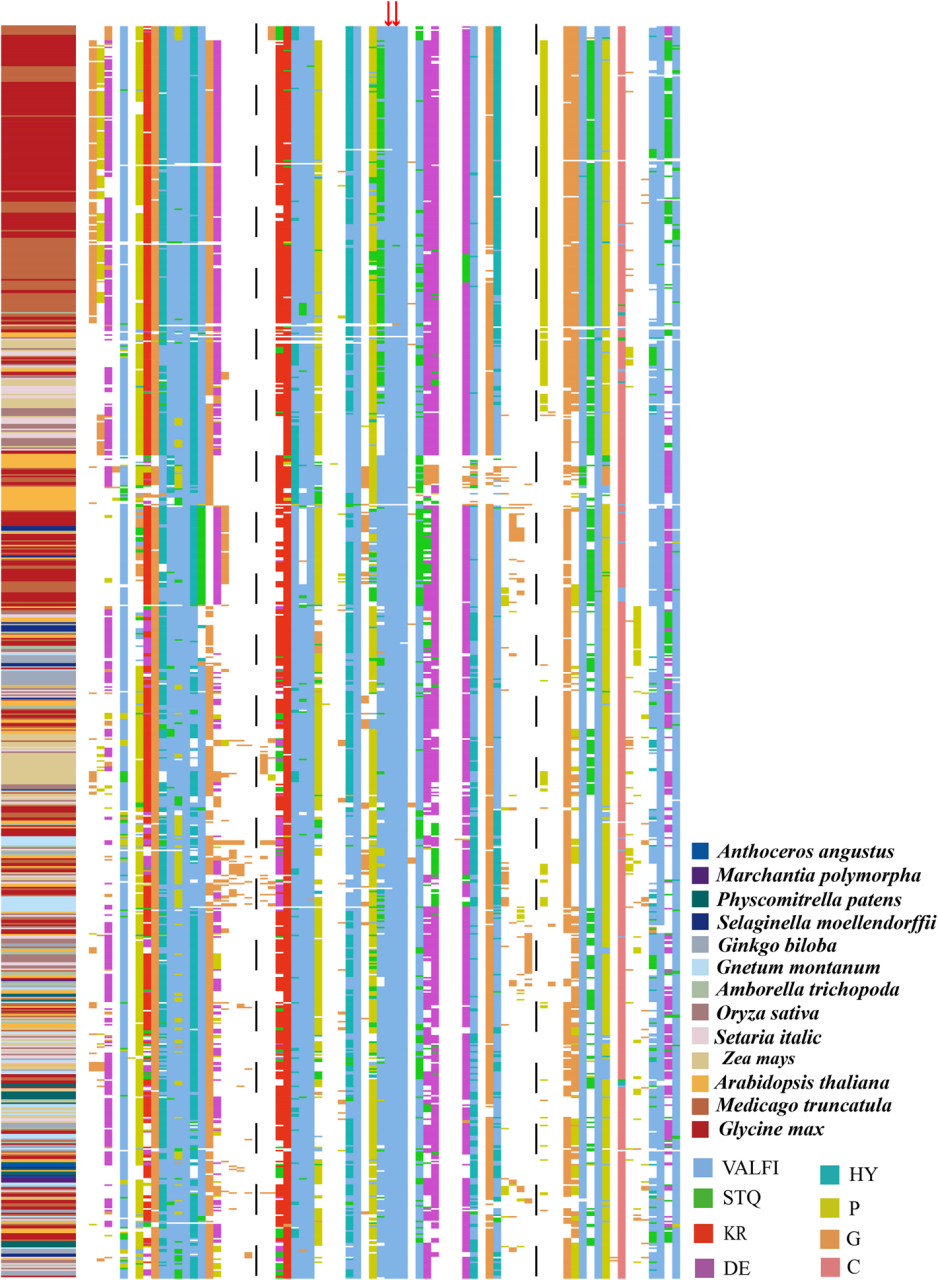
The conserved sequence of SAUR proteins from the thirteen plant species. 795 SAUR proteins from *Anthoceros angustus*, *Marchantia polymorpha*, *Physcomitrella patens*, *Selaginella moellendorffii*, *Ginkgo biloba*, *Gnetum montanum*, *Amborella trichopoda*, *Arabidopsis thaliana*, *Oryza sativa*, *Zea mays*, *Setaria italica*, *Glycine max* and *Medicago truncatula* were used to carry out multiple sequence alignment in Clustal Omega. The amino acid residues at SAUR domain (~ 60 amino acid residues) were labeled in different color. Red arrows indicated highly conserved leucine residues positions

**Fig. 3:**
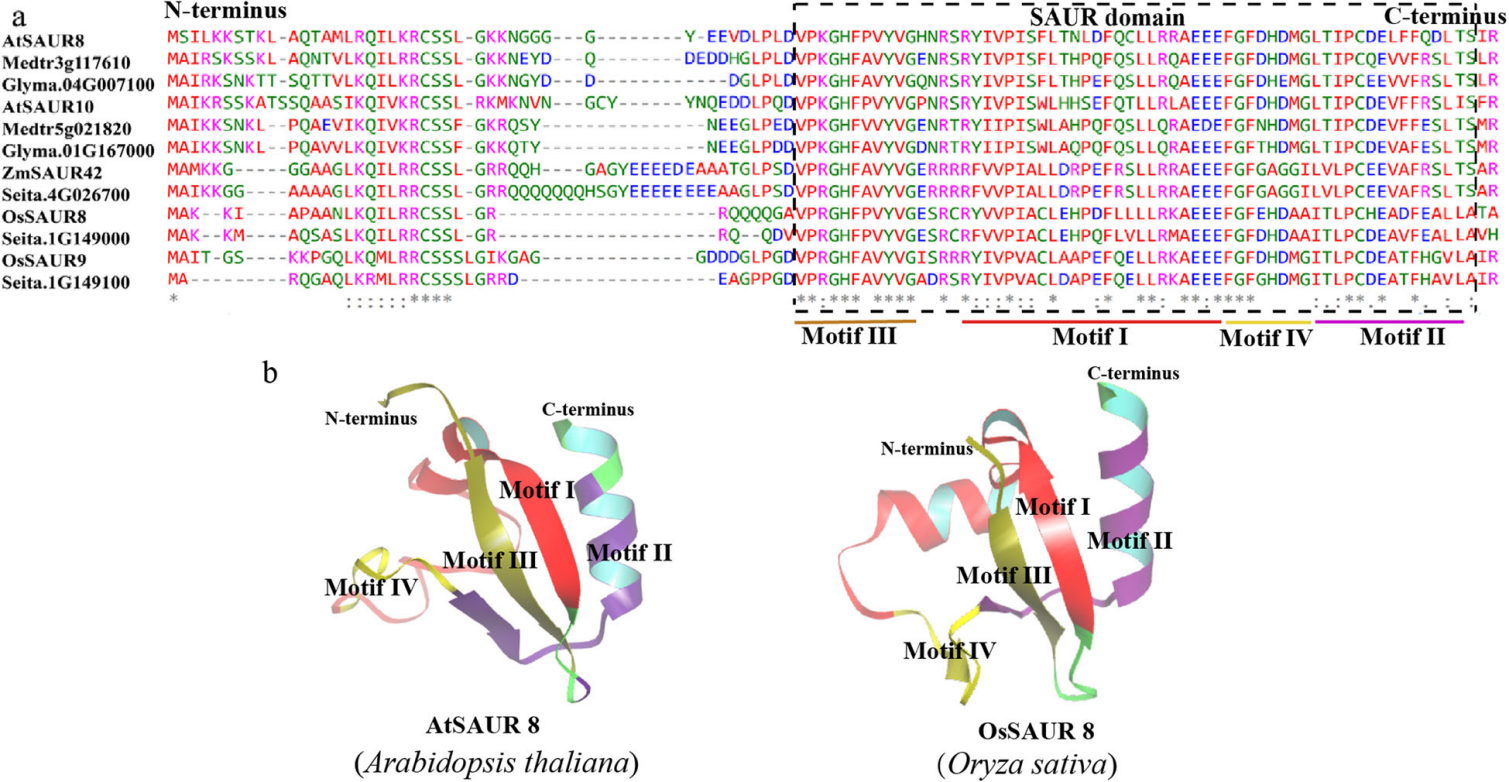
Sequence analysis and structure prediction of SAUR proteins in clade 2. **a** Multiple sequence alignment of SAUR proteins in clade 2 using Clustal Omega. Some nonconserved insertions of a few SAUR members were removed from the alignment. The dashed grey lines represented vacant sites (no amino acid residues) in multiple sequences alignment. The region of the SAUR domain was labeled in a black box. **b** The predicted tertiary structure of AtSAUR8 and OsSAUR8 using conserved domain only

Multiple sequence alignment of all SAUR proteins from *Anthoceros angustus*, *Marchantia polymorpha*, *Physcomitrella patens*, *Selaginella moellendorffii*, *Ginkgo biloba*, *Gnetum montanum*, *Amborella trichopoda*, *Arabidopsis thaliana*, *Oryza sativa*, *Zea mays*, *Glycine max*, *Medicago truncatula* and *Setaria italica* revealed that the sequences shared a common conserved core region of ~ 60 amino acid residues, which is regarded as the SAUR domain (Figs. 2, 3), consistent with previous reports [1, 6]. In addition, we observed that the majority of SAUR members in eudicotyledons contained a larger number of conserved amino acid sites compared with monocotyledons generally. Two leucine residues, which formed a hydrophobic core, were observed in almost all SAUR protein sequences, which suggested the residues play a critical role in the folding and basic functions of SAUR proteins (Fig. 2).

We identified five motifs within the conserved regions among the SAUR proteins from *Physcomitrella patens*, *Arabidopsis thaliana*, *Oryza sativa*, *Zea mays*, *Setaria italica*, *Glycine max* and *Medicago truncatula* (Supplementary Fig. 4). The majority of SAUR members contained motifs I to IV and ~ 20% of the SAUR sequences contained motif V. It is noteworthy that SAUR proteins of *Physcomitrella patens* only contained motifs I to III, indicating that SAUR proteins in higher plants have evolved novel motifs. However, the functions of these motifs are currently unknown and require further study.

Previous research showed that members of the AtSAUR10 subclade in clade 2 of the two trees induce cell elongation (Fig. 1, Supplementary Fig. 1) [28]. SAUR members that show a close homologous relationship with AtSAUR10 in clade 2 were highly conserved, although the N- and C-terminal regions were less conserved than the core region (Fig. 3a). The majority of SAUR proteins shown in Fig. 3a were predicted to localize in chloroplasts (Supplementary Table 2), which may be associated with the similarity of the N- or C-terminal regions. The conserved sequences of AtSAUR8 and OsSAUR8 displayed similar tertiary structures and similar motifs (Fig. 3b), as predicted using the SWISS-MODEL server [29] and subjected to molecular dynamics simulation using GROMACS2019.1 (http://manual.gromacs.org/2019.1/index.html) [30] for structure optimization: motif I developed a β-sheet, motif II formed a typical α-helix, motif III formed a β-sheet, and motif IV was the linker between motif I and motif II (Fig. 3b), which indicated that the functions of AtSAUR8 and OsSAUR8 may be similar. However, the precise functions of each motif in maintaining protein stability or activities require further study.

### Expression analysis of *SAUR* genes

**Fig. 4:**
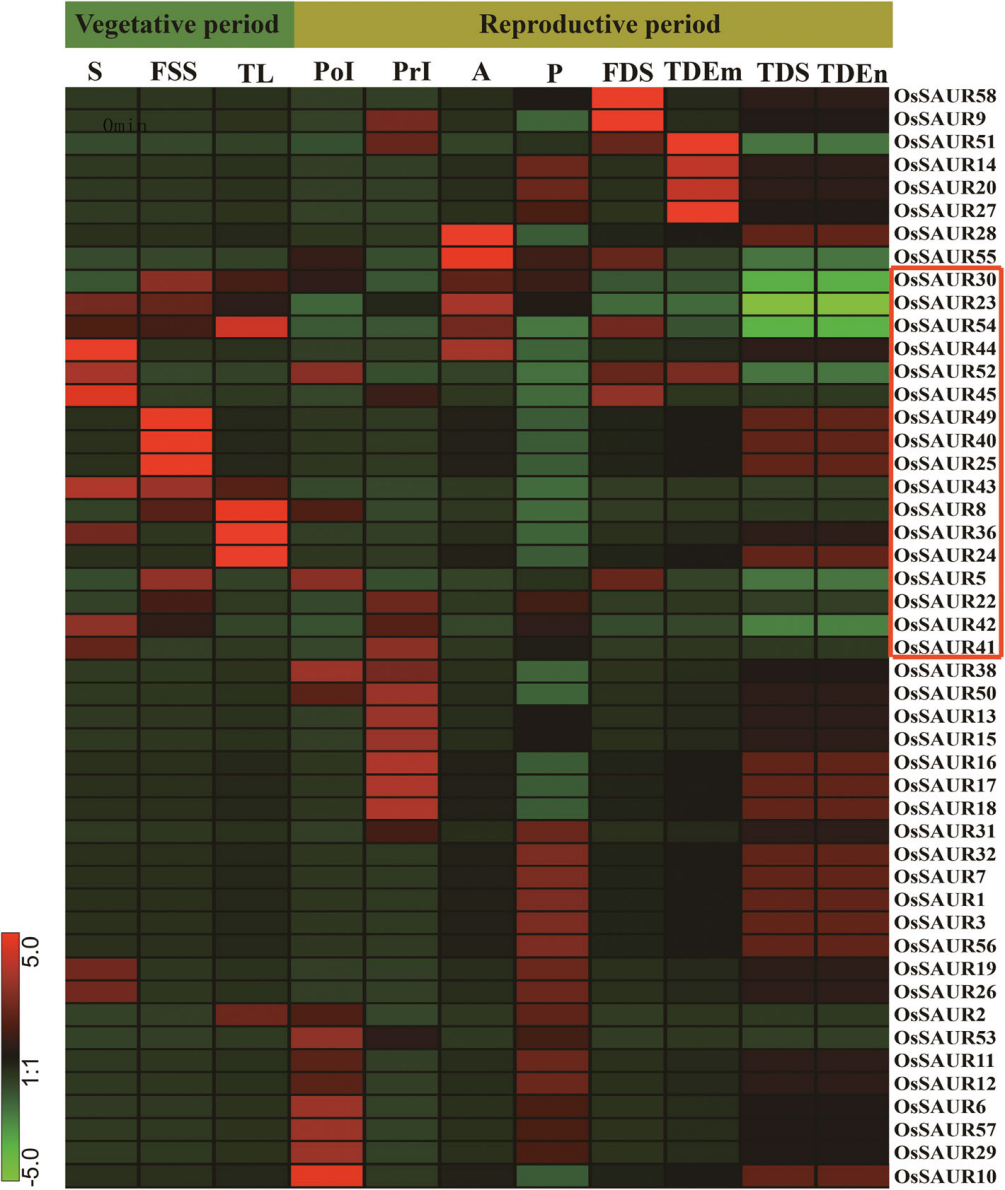
*OsSAURs* gene expression patterns from 11 different development tissues or organ systems by RNA-seq. S: Shoot; FSS: Four-leaf Stage Seedling; TL: Twenty-day Leaf; PoI: Post-emergence Inflorescence; PrI: Pre-emergence Inflorescence; A: Anther; P: Pistil; FDS: Five DAP Seed; TDEm: Twenty-five DAP Embryo; TDS: Ten DAP Seed; TDEn: Twenty-five DAP Endosperm; DAP: Days After Pollination

**Fig. 5:**
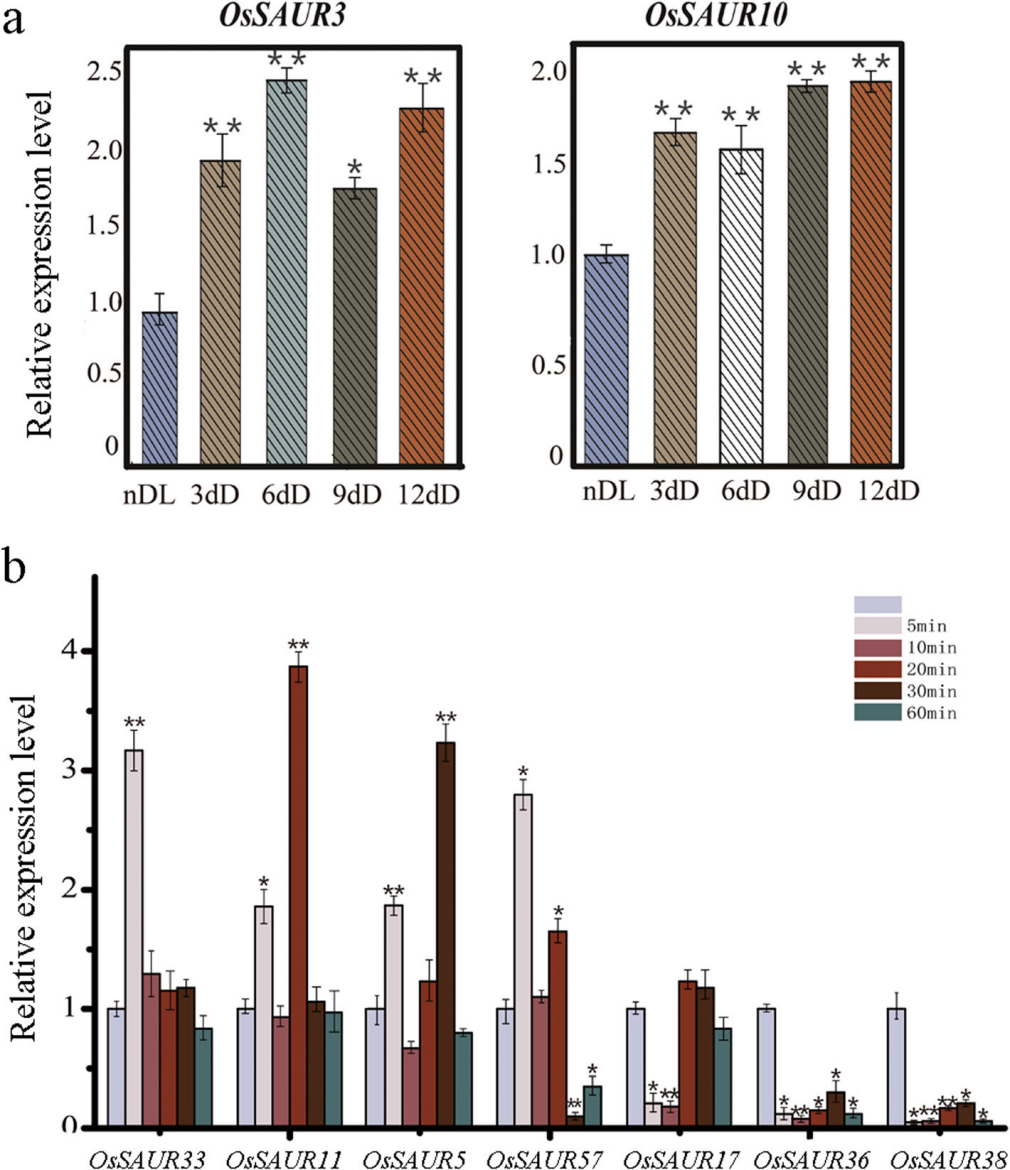
Expression analysis of small auxin-up RNA (*SAUR*) genes in rice. **a** Relative expression level of *OsSAUR3* and *OsSAUR10* in clade 6 of FastTree tree (clade 7 of IQ-TREE tree) from leaf blade of normal seedings and etiolated seedings under different days (ndL, n-day-old light-grown seedings, *n* = 3, 6, 9, 12; 3dD, 3-day-old etiolated seedings, etc.). **b** Relative expression level of the seven *OsSAUR* genes in rice seedlings treated by 10 μM IAA. The expression of *OsUBQ5* was used as an internal control. Data were mean ± SE from three biological replicates. * indicate statistically significant differences by student t-test: *P* < 0.05. ** *P* < 0.01

Expression pattern analysis is a useful tool for understanding gene functions. A comprehensive expression analysis was performed using RNA-seq data to explore expression profiles of *SAUR* genes in rice and soybean (Fig. 4, Supplementary Fig. 5). The expression level was estimated for all *OsSAUR* genes detected from 11 tissues and organs, except for eight genes (*OsSAUR39*, *OsSAUR37*, *OsSAUR4*, *OsSAUR48*, *OsSAUR47*, *OsSAUR46*, *OsSAUR33* and *OsSAUR21*) (Fig. 4). The majority of *OsSAUR* genes in clade 5 of the two trees were highly expressed in the vegetative phase but were weakly expressed in the reproductive phase, which showed a good agreement with their chloroplast localization (Figs. 1, 4, Supplementary Fig. 1, Supplementary Table 2). *OsSAUR20* and *OsSAUR27* of clade 8 of the two trees were expressed highly in the embryo at 25 days after pollination (DAP) (Figs. 1, 4, Supplementary Fig. 1), which suggested members of this clade are involved in embryo development in rice. Almost all *GmSAUR* genes in clade 3 of FastTree tree (clade 6 of IQ-TREE tree) showed an identical expression pattern, namely expressed highly in seeds within 25 ~ 42 days after flowering (DAF) and weakly in the pod shell and flower within 14 DAF (Fig. 1, Supplementary Figs. 1, 5). Four *GmSAUR* genes (*Glyma. 3 g259700*, *Glyma. 7 g051700*, *Glyma. 16 g020700* and *Glyma. 1 g137500*) in clade 4 of the two trees were predominantly expressed in the seed (Fig. 1, Supplementary Figs. 1, 3), which implied that members of this clade were involved in seed development. Compared with *OsSAUR* genes, a greater number of *GmSAUR* genes showed a similar expression pattern. In addition, based on digital gene expression libraries, we observed that the expression of many *OsSAUR* genes was induced by abiotic and biotic stresses, especially salt, drought and rice blast (Supplementary Fig. 6), which indicated that many *SAUR* genes mediate stress responses, although few studies have investigated such functions to date. Then we investigated the expression patterns of *OsSAUR* genes in clade 6 of FastTree tree (clade 7 of IQ-TREE tree) treated by dark. The results showed that *OsSAUR3* and *OsSAUR10* were highly expressed in etiolated seedlings (Fig. 5a). Jain et al. (2006) observed that the expression levels of *OsSAUR7* in the same clade were also significantly higher in etiolated seedlings than in normal seedlings [10], which indicated they play similar roles in light and hormone responses. These results indicated that the expression patterns of members of the same clade were often similar.

To clarify the response of *SAUR* genes to auxin, we also investigated the expression patterns of *OsSAURs* genes selected randomly from different clades in the leaf blade of rice seedlings treated with 10 μM IAA. The results revealed extremely diverse auxin-responsive expression patterns (Fig. 5b). Three genes (*OsSAUR33*, *OsSAUR11* and *OsSAUR5*) were induced rapidly at 5 min after auxin treatment, whereas three genes (*OsSAUR17*, *OsSAUR36* and *OsSAUR38*) were strongly inhibited within 5 min by exogenous auxin. Strangely, *OsSAUR57* was induced rapidly at 5 min and then was strongly suppressed at 30 min after 10 μM IAA treatment. These results showed that expression of *SAUR* genes was closely associated with exogenous auxin application time and indicated that *SAUR* genes play an extremely complex role in the auxin-mediated signal pathway.

### Functions of identified *SAUR* genes

**Fig. 6:**
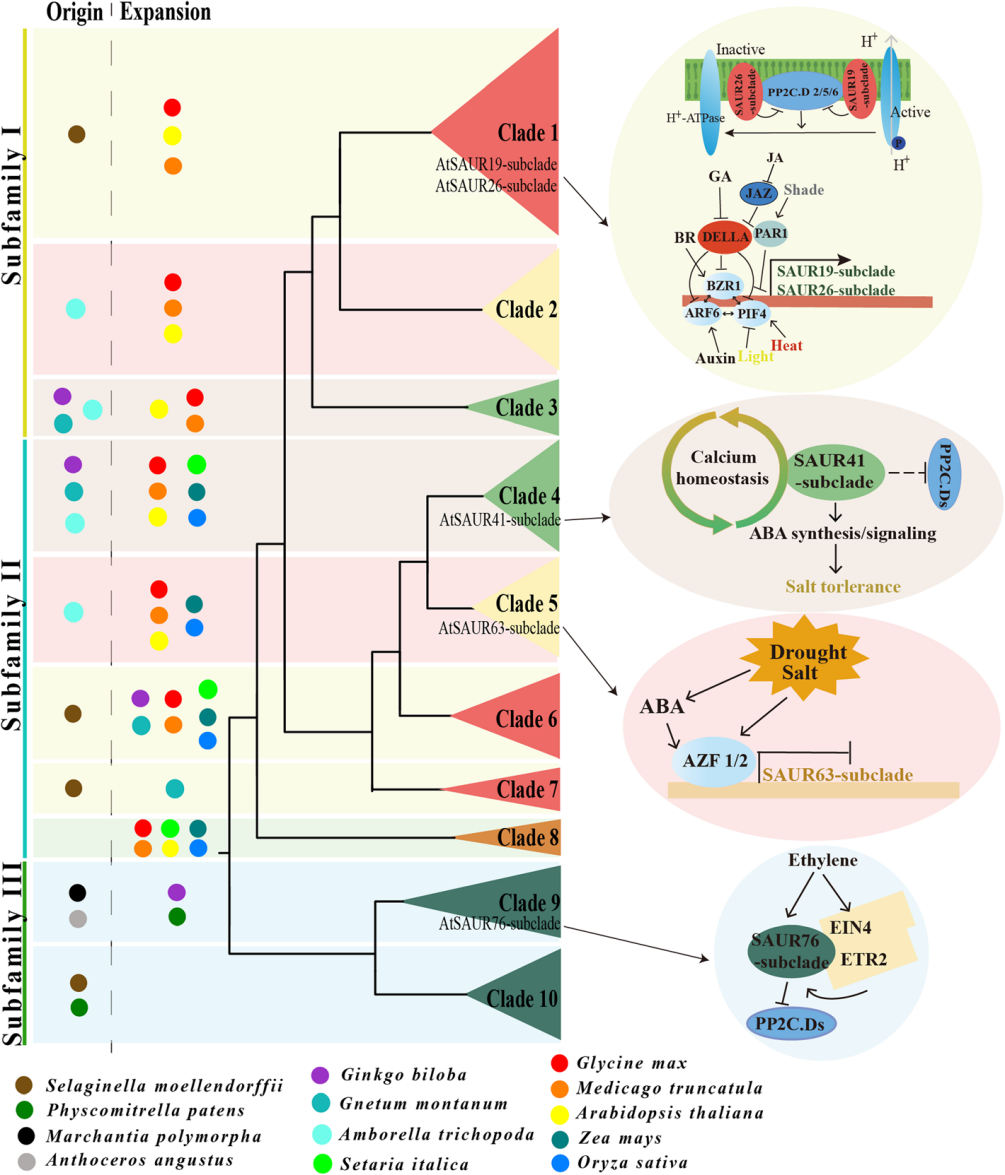
The simplified schema and functions of *SAUR* genes in plant evolution. During the process of plant evolution, *SAUR* gene family from bryophyta to anthophyta expended largely, and every clade experienced expansion at different degrees (left panel). Right panel of Fig. 6 showed the function of *SAUR* genes in response to hormonal and environmental signals: based on acid growth mediated by plasma membrane H^+^-ATPase, auxin-responsive SAUR proteins (AtSAUR 19- and AtSAUR 26-subclades in clade 1) activate plasma membrane H^+^-ATPase by inhibiting PP2C-D phosphatases to promote cell expansion [31]. The AtSAUR76-subclade in clade 9 mediates ethylene signaling via interaction with Ethylene Insensitive 4 (EIN4) and Ethylene Receptor 2 (ETR2) to promote plant growth [32]. The SAUR41-subclade is inducible by abscisic acid to modulate cell expansion and salt tolerance, and maintain calcium homeostasis [24]. Auxin Response Factor 6 (ARF6), Brassinazole Resistant1 (BZR1) and Phytochrome Interacting Factor 4 (PIF4) may directly regulate transcription of *AtSAUR19*-subclade and *AtSAUR26*-subclade independently or as a complex [33] sensing hormonal and environmental signals including auxin, brassinosteroids (BR), gibberellin (GA), jasmonic acid (JA), light, heat and shade [25, 28, 33–40]. *Arabidopsis* zinc-finger protein 1 (AZF1) and AZF2 in the ABA-response pathway can repress the expression of AtSAUR63 subclade [41]

To explore SAUR functions, we summarized the functions of all currently identified *SAUR* genes (Fig. 6, Supplementary Table. 3). Although SAUR proteins influence diverse aspects of plant growth and development, the molecular mechanisms could be summarized in relatively few processes. The AtSAUR19-subclade and AtSAUR26-subclade in clade 1 of the two trees inhibit PP2C-D phosphatases and then activate plasma-membrane H^+^-ATPase activity to promote cell expansion (Figs. 1, 6, Supplementary Fig. 1) [25, 31]. The AtSAUR76-subclade in clade 9 of the two trees mediates ethylene signaling via interaction with Ethylene Insensitive 4 (EIN4) and Ethylene Receptor 2 (ETR2) to promote plant growth (Figs. 1, 6, Supplementary Fig. 1) [32]. AtSAUR49 activates Senescence-Associated Receptor-Like Kinase (SARK)-mediated leaf senescence signaling by suppression of Senescence Suppressed Protein Phosphatase (SSPP) [19]. AtSAUR70 binds to calmodulin in a calcium-dependent manner in vivo [42], which is indicative of a link between auxin signaling and the second messenger Ca^2+^/calmodulin. *OsSAUR39* overexpression negatively regulates auxin biosynthesis and transport [43]. In addition, *AbSAUR1* overexpression significantly improves the overall yield of tropane alkaloids, which are regarded as anticholinergic drugs in the clinic, attaining a yield 3.55 times that of the control [44]. The researchers also observed that the genes in the same evolutionary lineage showed similar functions, and thus investigated the *SAUR* genes group as one unit, for example, the AtSAUR19-subclade and AtSAUR26-subclade in clade 1, AtSAUR10-subclade in clade 2, AtSAUR63-subclade in clade 5, AtSAUR41-subclade in clade 4, and AtSAUR76-subclade in clade 9 of the two trees (Figs. 1, 6, Supplementary Fig. 1).

## Discussion

In the process of adaptation to diverse terrestrial environments, plants have evolved a suite of hormones to respond to developmental and environmental signals [45]. Auxin signaling occurs later in evolution compared with cytokinin and ethylene, and was initially discovered in streptophyte algae [46, 47]. But none SAUR homolog was found in streptophyte algae and other alga, speculating that SAUR proteins appeared relatively later. *SAUR* genes of bryophyte were first identified in *Physcomitrella patens* [26]. In the present study we first identified *SAUR* genes from the reference genome sequences for *Anthoceros angustus* (3), *Marchantia polymorpha* (5), *Selaginella moellendorffii* (15), *Ginkgo biloba* (42), *Gnetum montanum* (37), *Setaria italica* (58), *Mimosa pudica* (62), *Cercis canadensis* (48) *Medicago truncatula* (141), and *Glycine max* (236). So large expansion of *SAUR* family from bryophyta to anthophyta should contribute to greatly balance developmental and environmental signals, which is critical for blossom of terrestrial plant (Fig. 6). Compared with the number of *SAUR* genes in other dicotyledons, a greater number are present in the genomes of soybean and *Medicago truncatula* (Figs. 1, 6, Supplementary Fig. 1). Combined with *SAUR* gene synteny analysis between Fabaceae and *Arabidopsis thaliana* (Supplementary Fig. 3), which suggested that *SAUR* family expansion might be related to whole genome duplications in Fabaceae plants [48]. We observed less expansion in monocotyledons than in eudicotyledons in general, which was also verified by previous studies on *SAUR* gene number in plant species such as cotton (145) [11], citrus (70) [12], bamboo (44) [13], ramie (71) [14] and watermelon (65) [15] (Supplementary Fig. 7).

Understanding protein structure is vital to determining the function of a protein and its interaction with other proteins. Prediction of protein structure has profound theoretical and practical influences on biological research [49]. In the present study, we identified five conserved motifs among SAUR proteins and observed that the majority of SAURs contain motifs I to IV and ~ 20% of SAUR family members contain motif V (Supplementary Fig. 4). This result is similar to predictions for cotton [11]. The tertiary structures of AtSAUR8 and OsSAUR8 showed that motifs I to IV generated a similar structure (Fig. 3b), however, the fine functions of each motif in maintaining protein stability and activities require further study. In addition, we observed that two leucine residues in motif I located at the hydrophobic core positions were present in almost all SAUR proteins (Fig. 2), and might be involved in protein interactions, such as with PP2C.D [31]. However, their critical role in the biochemical function of SAUR proteins needs to be confirmed by conducting point-mutation genetic experiments in the future.

The functions of SAUR proteins remain mysterious despite previous notable breakthroughs (Fig. 6, Supplementary Table 3). We examined the expression level of seven randomly chosen *OsSAUR* genes in response to IAA treatment. The expression level of four genes was upregulated, whereas three genes were downregulated, at 5 min after IAA treatment (Fig. 5b). Digital gene expression analysis also suggested that many *OsSAUR* genes are responsive to salt, drought and rice blast (Supplementary Fig. 4). The function of *SAUR* genes in resistance to rice blast is worth further study. A recent study reported that *SlSAUR69* enhances the sensitivity of tomato fruit to ethylene through repression of polar auxin transport to influence the unripening-to-ripening transition, which revealed that SlSAUR69 mediates crosstalk between auxin and ethylene [50]. The AtSAUR26 subfamily, which was identified as a QTL for growth thermo-responsiveness, shows temperature-related natural polymorphisms that influence the thermo-responsiveness of plant architecture for adaptation to the local temperature [25]. These results indicated that *SAUR* genes from different clades participate in a multitude of processes involved in plant growth and stress response.

The present expression analysis of *SAUR* genes showed that the expression pattern was similar among members of the same clade. For example, *OsSAUR3*, *OsSAUR10* and *OsSAUR7* of clade 6 of FastTree tree (clade 7 of IQ-TREE tree) were highly expressed in etiolated seedlings (Fig. 5a) [10]. The majority of *OsSAUR* genes in clade 5 were highly expressed in the vegetative phase and localized in chloroplasts (Fig. 4, Supplementary Table 2). Four *GmSAUR* genes (*Glyma.3 g259700*, *Glyma.7 g051700*, *Glyma.16 g020700* and *Glyma.1 g137500*) in clade 4 were predominantly expressed in the seed (Fig. 1, Supplementary Fig. 1, Supplementary Fig. 5). These results indicated that genes in the same evolutionary lineage show functional similarity, which is confirmed by functional experiments. For example, the AtSAUR19-subclade and AtSAUR26-subclade in clade 1 are involved in so-called “acid growth” by invoking plasma membrane H^+^-ATPase activation [51], and the AtSAUR41-subclade in clade 4 maintains calcium homeostasis and modulates drought and salt tolerance (Figs. 1, 6, Supplementary Fig. 1).

## Conclusions

In this study, we present a phylogenetic framework for the *SAUR* gene family from thirteen plant species. The *SAUR* gene family shows notable expansion from bryophyta to anthophyta, particularly in Fabaceae plants, and can be divided into three subfamilies and 10 clades (Fig. 1, Supplementary Fig. 1, Supplementary Datasheets 1, 2). Multiple sequence alignment and motif and protein structure prediction indicate that SAUR proteins are highly conserved (Figs. 2, 3). SAUR proteins participate in a multitude of processes involved in plant growth and development. Although *SAUR* genes from different clades show diverse expression patterns, members of the same clade show functional similarity. Future elucidation of the functions of *SAUR* family members will require a concerted effort by adoption of diverse approaches, including molecular genetic analysis based on an evolutionary lineage as the study unit.

## Methods

### Identification and sequence analysis of *SAUR* family members

SAUR protein sequences of *Arabidopsis thaliana* were used as queries in a BLAST search (score > 50, E-value < 0.01) of the Phytozome 12 database (https://phytozome.jgi.doe.gov). Sequences for SAUR members from *Arabidopsis thaliana*, *Glycine max*, *Medicago truncatula*, *Marchantia polymorpha*, *Physcomitrella patens*, *Selaginella moellendorffii* and *Amborella trichopoda* were downloaded*.* Sequences were obtained for SAUR members in *Oryza sativa*, *Zea mays* and *Setaria italica* with the same approach but using SAUR protein sequences of *Oryza sativa* as queries. We used SAUR protein sequences of *Arabidopsis thaliana* as queries to search SAUR members identification of *Anthoceros angustus*, *Gnetum montanum*, *Ginkgo biloba*, *Mimosa pudica* and *Cercis canadensis* via local blastp (score > 50, E-value < 0.01) of ncbi-blast-2.10.1+ (ftp://ftp.ncbi.nlm.nih.gov/blast/executables/blast+/LATEST/). Protein sequences of these five species were downloaded from the DRYAD website (https://datadryad.org/stash/dataset/doi:10.5061/dryad.msbcc2ftv, https://datadryad.org/stash/dataset/doi:10.5061/dryad.0vm37) and the GigaScience GigaDB repository (https://db.cngb.org/search/literature/27871309, http://gigadb.org/dataset/101044, http://gigadb.org/dataset/101049) respectively. *SAUR* members identification of chlorophyte species (*Chlamydomonas reinhardtii*, *Dunaliella salina*, *Volvox carteri*, *Coccomyxa subellipsoidea* C-169, *Micromonas pusilla* CCMP1545, *Micromonas* sp. RCC299 and *Ostreococcus lucimarinus*) and streptophyte algae species (*Spirogloea muscicola*, *Mesotaenium endlicherianum*, *Mesostigma viride* and *Chlorokybus atmophyticus*) was carried out via a BLAST search using SAUR protein sequences of *Arabidopsis thaliana* as queries in Phytozome 12 database (https://phytozome.jgi.doe.gov) and NCBI database (https://www.ncbi.nlm.nih.gov) respectively. Seven hundred ninety-five SAUR protein sequences from *Anthoceros angustus*, *Marchantia polymorpha*, *Physcomitrella patens*, *Selaginella moellendorffii*, *Ginkgo biloba*, *Gnetum montanum*, *Amborella trichopoda*, *Arabidopsis thaliana*, *Oryza sativa*, *Zea mays*, *Glycine max*, *Medicago truncatula* and *Setaria italica* were aligned with clustalx 2.1 [52] and inspected manually with Jalview [53]. Conserved regions of *SAUR* gene family (~ 60 amino acids) were greatly aligned and there was local homology in non-conserved regions. Some extremely gapped positions were manually removed, and nearly all non-conserved regions with local homology were retained. Finally obtained sequence alignment was used to constructed FastTree and IQ-TREE tree. Physicochemical parameters of SAUR proteins were analyzed using ProtParam (http://web.expasy.org/protparam) [54]. The Plant-mPLoc online tool (http://www.csbio.sjtu.edu.cn/bioinf/plant-multi/) [27] was used to predict the subcellular localization of SAUR proteins.

### Phylogenetic analysis of SAUR family

Phylogenetic analysis of the multiple sequence alignment of SAUR proteins was conducted. Phylogenetic trees were constructed using FastTree v2.1 [55] with the JTT + CAT model and IQ-TREE v2.0.6 [56] with the JTT + R9 model respectively. Support for each node was assessed by performing a bootstrap analysis with 1000 replicates.

### Multiple sequence alignment and motif prediction of SAUR proteins

Protein sequences of SAUR family members were used to generate a multiple sequence alignment and visualization analyses using Clustal Omega (https://www.ebi.ac.uk/Tools/msa/clustalo) [57] and Jalview [53], respectively. Only the conserved portion of each sequence was retained, which was then applied for motif prediction using MEME (http://meme-suite.org).

### Plant materials and growth conditions

Asian cultivated rice (*Oryza sativa subsp. japonica* cv. Nipponbare, of which was completed the genome sequencing in 2004) was used in this study. Collection of this rice variety was complied with the institutional and national guidelines in China, and seeds were stored in our lab. Rice seeds were treated and grown as described previously [58]. For auxin treatment, the 2-week-old seedlings were incubated in Yoshida nutrition solution [59] containing 10 μM IAA and then sampled at 5, 10, 15, 30 and 60 min, respectively. For dark treatment, the seedlings were grown in the plant incubator without light all the time and sampled at 3, 6, 9 and 12 day, respectively. Control (CK) plants were all grown in the plant incubator with normal condition.

### Quantitative real-time PCR analysis

Total RNA was extracted with TRIzol Reagent from leaf blade of rice seedlings grown under the normal condition and different treatments. For plants, growing parts (such as root and leaf) often can produce a large amount of auxin to maintain the needs of plant growth. The genes *OsSAUR3* and *OsSAUR10* were chosen to examined to respond to light. So, RNA was used from leaf blade to run qRT-PCR. The cDNA was synthesized using the PrimeScript RT Reagent Kit with gDNA Eraser (Takara, Kyoto, Japan). PCR amplifications were performed using the TransStart Tip Green qPCR SuperMix (TransGen Biotech, Beijing, China) on the CFX96™ Real-Time PCR Detection System (Bio-Rad, Hercules, CA, USA). Gene-specific primers used in the experiments are listed in Supplementary Table 4. Three biological replicates were performed for each reaction. Relative gene expression levels were calculated from the qRT-PCR data using the 2^−△△*C*t^ method [60].

### Expression analysis based on RNA-seq and digital gene expression data

The expression data were derived from the Rice Genome Annotation Project (http://rice.plantbiology.msu.edu/expression.shtml) and Soybase Database (https://www.soybase.org/soyseq/) [61]. These data were gene-wise normalized within different plant tissues and hierarchically clustered on the basis of Pearson correlation coefficients using the weighted pair group method with averaging linkage with Genesis (v1.7.6) software [62]. Digital gene expression data was visualized in Genesis using white and black color to represent “unexpressed” and “expressed” of genes.

### Protein tertiary structure prediction and optimization

Prediction of the tertiary structure prediction of SAUR proteins was performed using SWISS-MODEL (https://swissmodel.expasy.org/) [29]. Sequence identities of AtSAUR8 and OsSAUR8 with templates were 23.40 and 21.15%, respectively. The predicted structures were subjected to MD simulation using GROMACS 2019.1 software package (http://manual.gromacs.org/2019.1/index.html) with the Charmm 27 force field and TIPS3P water model in a box large enough to enclose the whole protein, and then subjected to energy minimization with 50,000 steps of steepest descent. The minimized structure was equilibrated with NVT and NPT simulation in turn. Finally, we carried out 1 ns long NVT MD simulation and structural parameters like RMSD converged after 800 ps. Analysis of RMSD and Ramachandran plot for structure optimization were shown in Supplementary Fig. 8. Visualization of protein structures was conducted with NOC 3.01 (http://noch.sourceforge.net/).

### Gene synteny analysis between Fabaceae plants and *Arabidopsis thaliana*

Genome assembly sequence and gene annotation of *Arabidopsis thaliana, Glycine max* and *Medicago truncatula* were download from Ensembl Plants database (http://plants.ensembl.org/index.html). The genes synteny analysis was built with MCScanX [63] program in TBtools [64]. Synteny relationship of *SAUR* genes was highlighted with blue lines.

## Supplementary Information

**Additional file 1: Supplementary Datasheet S1**. Maximum likelihood phylogenetic tree constructed by FastTree v2.1 of small auxin-up RNAs (*SAURs*) containing detailed names from the thirteen plant species.

**Additional file 2: Supplementary Datasheet S2**. Maximum likelihood phylogenetic tree constructed by IQ-TREE v2.0.6 of small auxin-up RNAs (*SAURs*) containing detailed names from the thirteen plant species.

**Additional file 3: Supplementary Table 1**. Identified SAUR protein sequences from *Anthoceros angustus*, *Marchantia polymorpha*, *Physcomitrella patens*, *Selaginella moellendorffii*, *Ginkgo biloba*, *Gnetum montanum*, *Amborella trichopoda*, *Arabidopsis thaliana*, *Oryza sativa*, *Zea mays*, *Setaria italica, Mimosa pudica*, *Cercis canadensis, Glycine max*, and *Medicago truncatula*.

**Additional file 4: Supplementary Table 2**. The length, isoelectric point and subcellular location of small auxin-up RNA (SAUR) proteins from the thirteen plant species.

**Additional file 5: Supplementary Table 3**. The functions of all identified *SAUR* genes.

**Additional file 6: Supplementary Table 4**. Gene-specific primers used in this study.

**Additional file 7: Supplementary Fig. 1**. Maximum likelihood phylogenetic tree constructed by IQ-TREE of the *SAUR* gene family from the thirteen plant species.

**Additional file 8: Supplementary Fig. 2**. Maximum likelihood phylogenetic tree constructed by FastTree of the *SAUR* gene family in *Arabidopsis thaliana*, *Mimosa pudica*, *Cercis canadensis, Medicago truncatula* and *Glycine max*.

**Additional file 9: Supplementary Fig. 3**. Gene synteny analysis between Fabaceae plants and *Arabidopsis thaliana*.

**Additional file 10: Supplementary Fig. 4**. The five conserved motifs of SAUR proteins from seven plant species containing *Arabidopsis thaliana*, *Oryza sativa*, *Zea mays*, *Glycine max*, *Medicago truncatula*, *Setaria italica*, *Physcomitrella patens*.

**Additional file 11: Supplementary Fig. 5**. Expression profiles of small auxin-up RNA (*SAUR*) genes in soybean (*Glycine max*).

**Additional file 12: Supplementary Fig. 6**. Digital gene expression of *OsSAURs* from different developmental stage of rice varieties under diverse stress conditions.

**Additional file 13: Supplementary Fig. 7**. The number of identified *SAUR* genes from genomes annotated monocotyledons and dicotyledons.

**Additional file 14: Supplementary Fig. 8**. Analysis of structural parameter RMSD and Ramachandran plot for structure optimization.

## Data Availability

The datasets supporting the conclusions of this article are included within the article and its supplementary information files. The data of the multiple sequence alignment used for FastTree and IQ-TREE trees was available in TreeBASE Web (http://purl.org/phylo/treebase/phylows/study/TB2:S26122).

## Abbreviations

*SAUR*: Small auxin-up RNA
*GH3*: Gretchen hagen 3
*Aux/IAA*: Auxin/indoleacetic acid
qRT-PCR: Quantitative real-time PCR
DGE: Digital gene expression
ML: Maximum likelihood
PP2C: Type 2C protein phosphatase
EIN: Ethylene insensitive
ETR: Ethylene receptor
SARK: Senescence-associated receptor-like kinase
SSPP: Senescence suppressed protein phosphatase
DAP: Days after pollination
DAF: Days after flowering
CK: Cytokinin
QTL: Quantitative trait locus
PM H^+^-ATPases: Plasma membrane H^+^-ATPases
ABA: Abscisic acid
BR: Brassinosteroids
GA: Gibberellin
JA: Jasmonic acid
AZF: Arabidopsis zinc-finger protein
ARF: Auxin response factor
BZR: Brassinazole resistant
PIF: Phytochrome interacting factor
S: Shoot
FSS: Four-leaf stage seedling
TL: Twenty-day leaf
PoI: Post-emergence inflorescence
PrI: Pre-emergence inflorescence
A: Anther
P: Pistil
FDS: Five DAP seed
TDEm: Twenty-five DAP Embryo
TDS: Ten DAP seed
TDEn: Twenty-five DAP endosperm
WGD: Whole genome duplication
