# Supplementary material for: Genome-wide identification and characterization of small auxin-up RNA (SAUR) gene family in plants: evolution and expression profiles during normal growth and stress response
Source: BMC Plant Biol. 2021 Jan 6;21:4. doi: 10.1186/s12870-020-02781-x (PMC7789510; doi:10.1186/s12870-020-02781-x)
Supplement: Supplementary file 1 — Additional file 1: Supplementary Datasheet S1. Maximum likelihood phylogenetic tree constructed by FastTree v2.1 of small auxin-up RNAs (SAURs) containing detailed names from the thirteen plant species. [file 12870_2020_2781_MOESM1_ESM.pdf]

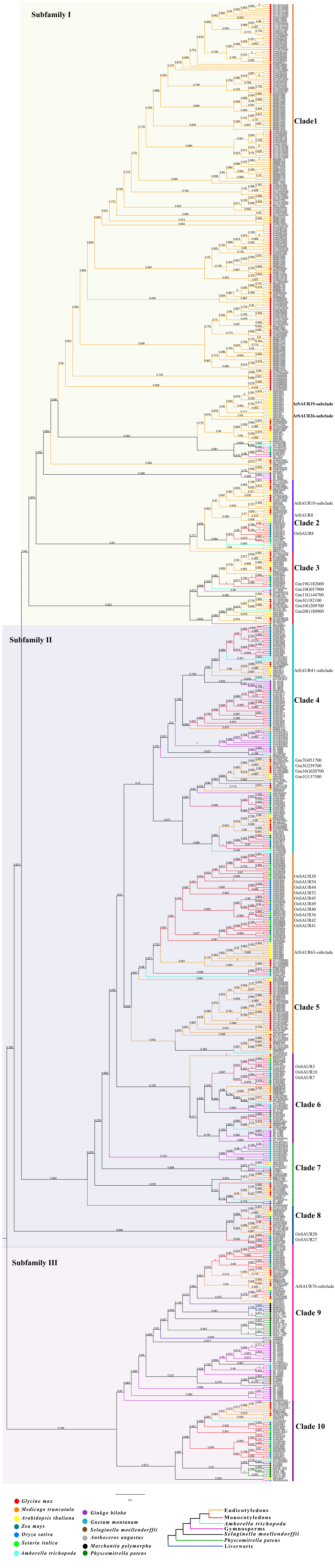

Subfamily I
Subfamily II
Subfamily III
Clade1
AtSAUR19-subclade
AtSAUR26-subclade
AtSAUR10-subclade
AtSAUR8
Clade 2
OsSAUR8
Clade 3
Gm19G182600
Gm10G057900
Gm13G144700
Gm3G182100
Gm10G209700
Gm20G180900
AtSAUR41-subclade
Clade 4
Gm7G051700
Gm3G259700
Gm16G020700
Gm1G137500
OsSAUR30
OsSAUR54
OsSAUR44
OsSAUR52
OsSAUR45
OsSAUR49
OsSAUR40
OsSAUR36
OsSAUR42
OsSAUR41
AtSAUR63-subclade
Clade 5
OsSAUR3
OsSAUR10
OsSAUR7
Clade 6
Clade 7
Clade 8
OsSAUR20
OsSAUR27
AtSAUR76-subclade
Clade 9
Clade 10
4.0
Glycine max
Medicago truncatula
Arabidopsis thaliana
Zea mays
Oryza sativa
Setaria italica
Amborella trichopoda
Ginkgo biloba
Gnetum montanum
Selaginella moellendorffii
Anthoceros angustus
Marchantia polymorpha
Physcomitrella patens
Eudicotyledons
Monocotyledons
Amborella trichopoda
Gymnosperms
Selaginella moellendorffii
Physcomitrella patens
Liverworts
