# Supplementary figures and images for: Genome-wide identification and characterization of small auxin-up RNA (SAUR) gene family in plants: evolution and expression profiles during normal growth and stress response
Source: BMC Plant Biol. 2021 Jan 6;21:4. doi: 10.1186/s12870-020-02781-x (PMC7789510; doi:10.1186/s12870-020-02781-x)

## Subfamily I

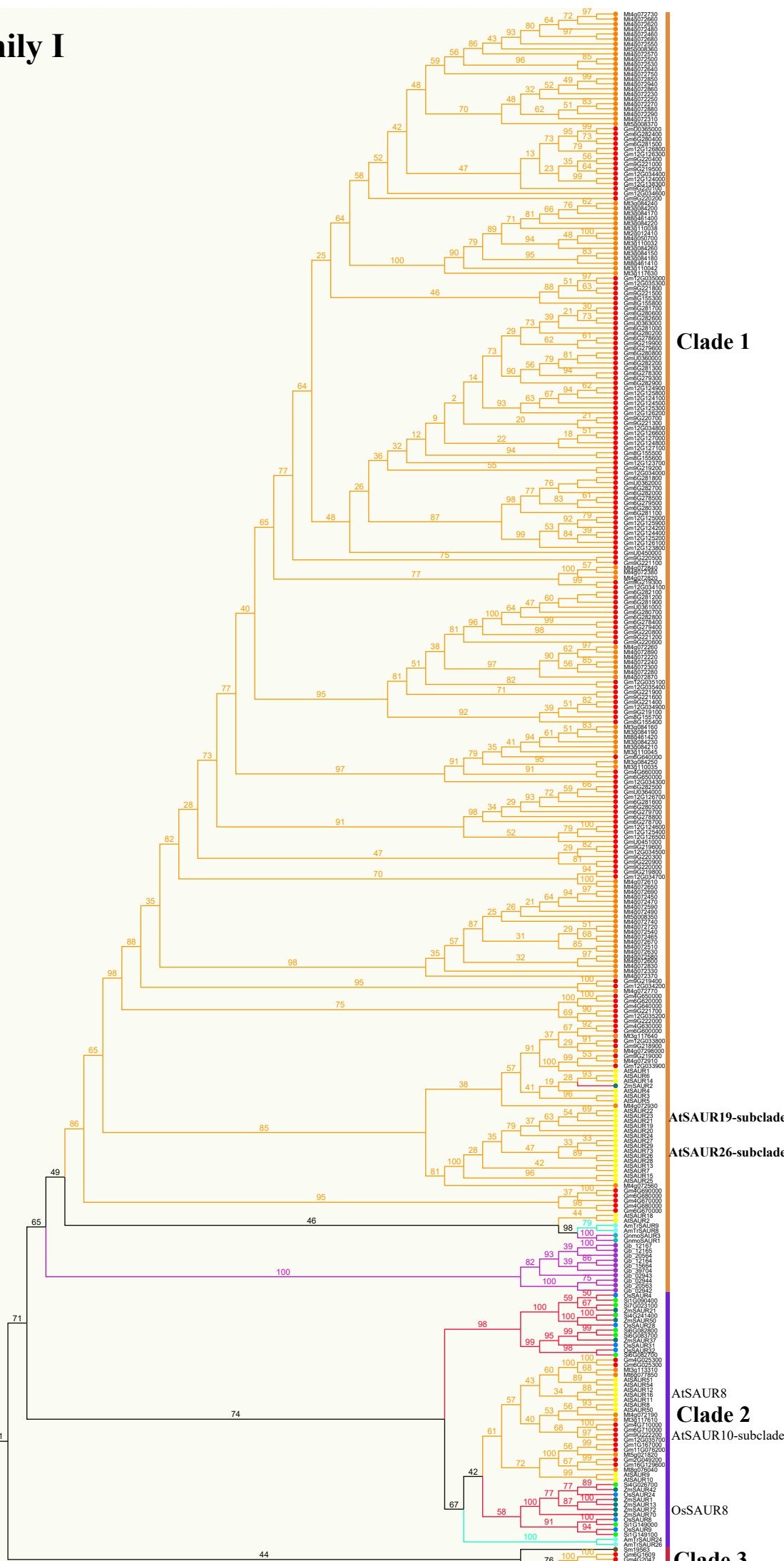

## Subfamily II

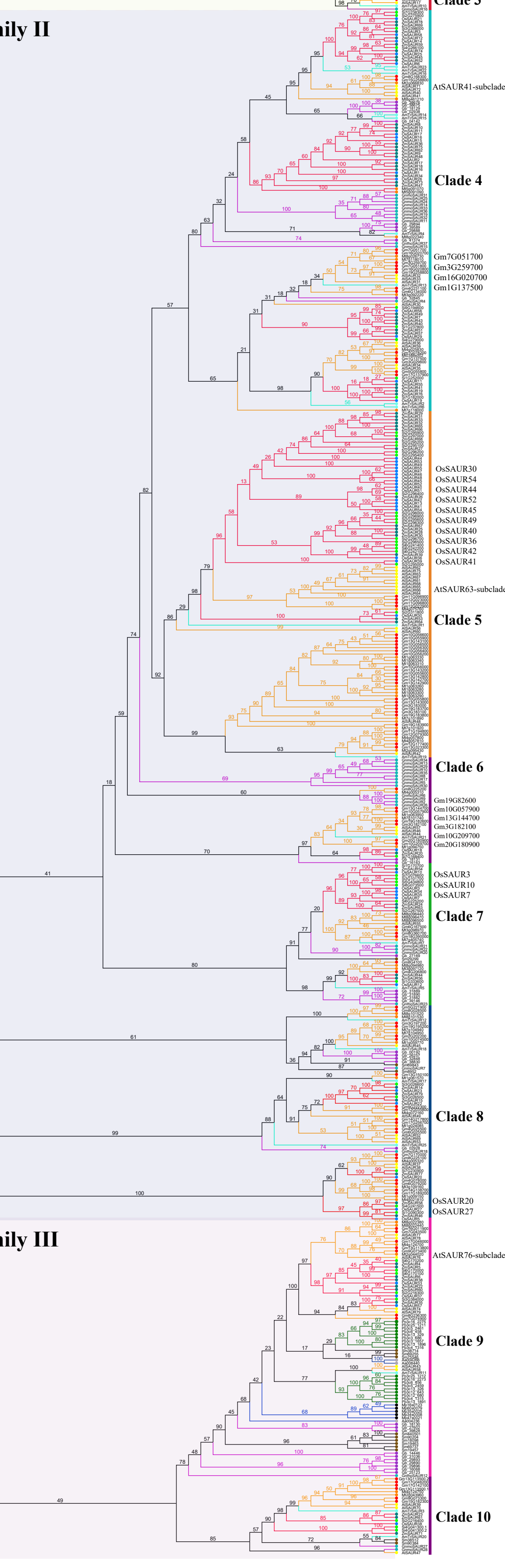

### Subfamily III

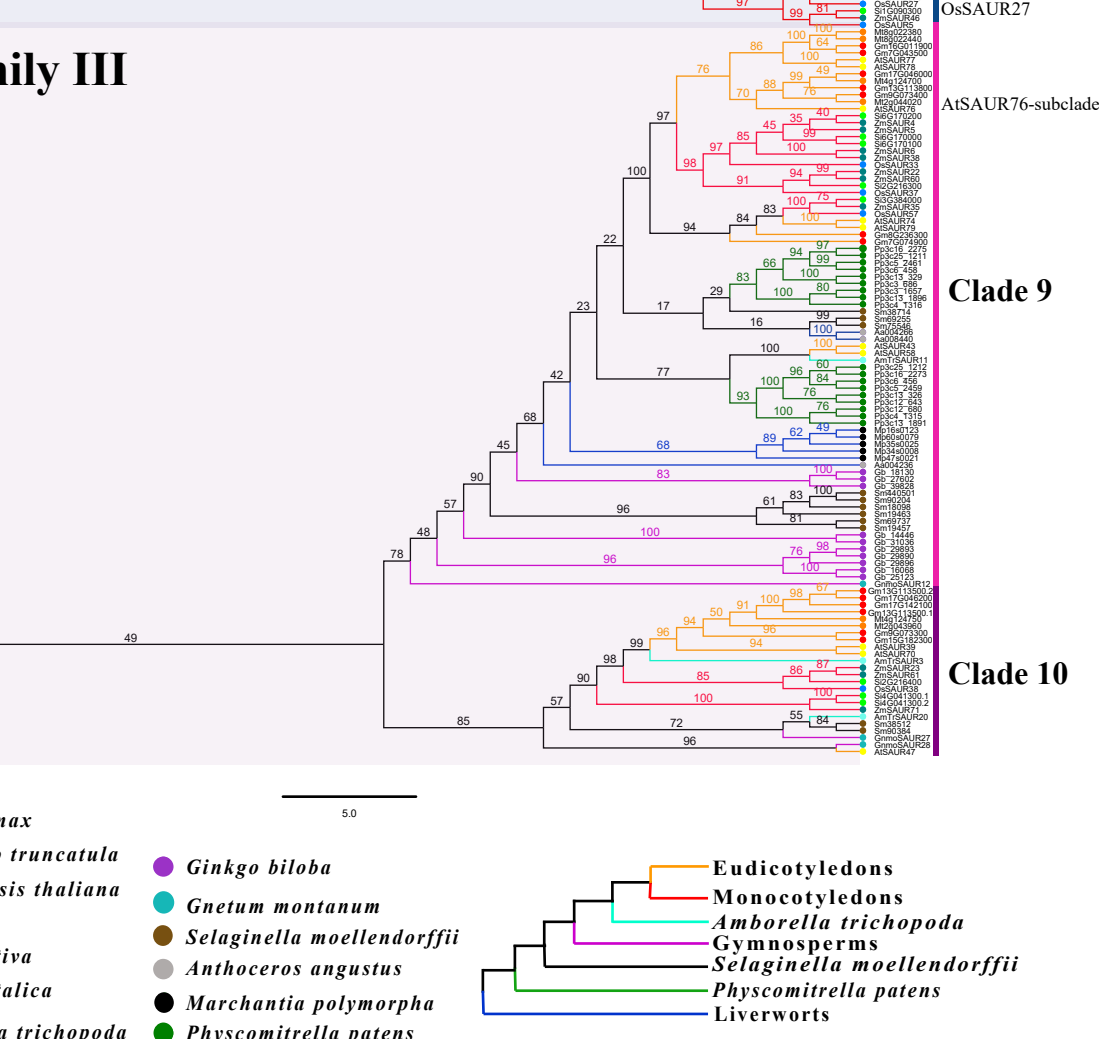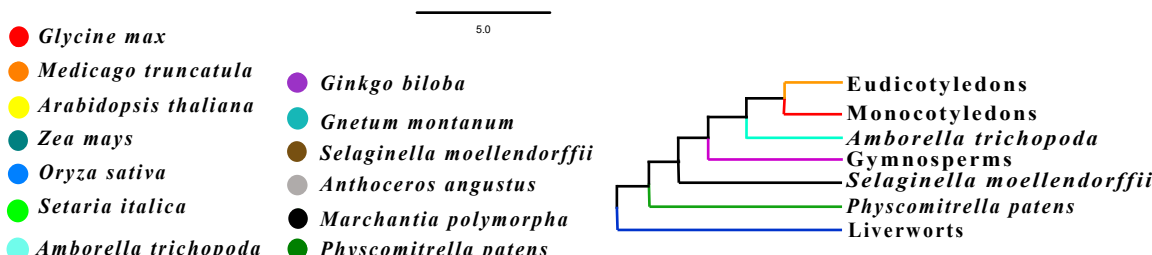

Supplement: Supplementary file 2 — Additional file 2: Supplementary Datasheet S2. Maximum likelihood phylogenetic tree constructed by IQ-TREE v2.0.6 of small auxin-up RNAs (SAURs) containing detailed names from the thirteen plant species. [file 12870_2020_2781_MOESM2_ESM.pdf]
