# Supplementary material for: Genome-wide identification and characterization of small auxin-up RNA (SAUR) gene family in plants: evolution and expression profiles during normal growth and stress response
Source: BMC Plant Biol. 2021 Jan 6;21:4. doi: 10.1186/s12870-020-02781-x (PMC7789510; doi:10.1186/s12870-020-02781-x)
Supplement: Supplementary file 5 — Additional file 5: Supplementary Table 3. The functions of all identified SAUR genes. [file 12870_2020_2781_MOESM5_ESM.docx]

Supplementary Table 3. The functions of all identified *SAUR* genes.

| Gene  Name | Gene ID  (Cloned) | Expression Pattern | Function  (Subcellular location) | References |
| --- | --- | --- | --- | --- |
| *AtSAUR36* | At2g45210  (Yes) | In senescing leaves, and induced by IAA, but repressed by GA | AtSAUR36 promotes leaf senescence. | (Hou et al., 2013; Stamm and Kumar, 2013) |
| *AtSAUR63* | At1g29440  (Yes) | In hypocotyls, cotyledons, petioles, young rosette leaves, the apical portion of the inflorescence stem, flowers. | AtSAUR63 promotes auxin-stimulated organ elongation including hypocotyls, stamen filaments, petals, hypocotyl, inflorescence stems.  (SAUR63 located in the plasma membrane, soluble and membrane fractions) | (Chae et al., 2012) |
| *AtSAUR76* | At5g20820  (Yes) | In very young anthers. | AtSAUR76 partially reduce ethylene sensitivity of ETR2 and promote plant growth in *Arabidopsis*.  (SAUR76 located in the nucleus, cytoplasm and plasma membrane) | (Markakis et al., 2013; Li et al., 2015) |
| *AtSAUR50* | At4g34760  (Yes) | In the hypocotyls, cotyledons, rosette leaves, cauline leaves, flower and silique, and can be repressed by dark-to-light transition. | AtSAUR50 is critical for differential light regulation of the development of cotyledons and hypocotyls. *SAUR 50* expression level directly is regulated by PIF and Auxin. | (Spartz et al., 2014; Sun et al., 2016) |
| *AtSAUR32*  (*AAM1*) | At2g46690  (Yes) | On the inner side of the apical hook. | AtSAUR32 is involved in the apical hook and hypocotyls developments.  (SAUR32 located in the nucleus) | (Park et al., 2007) |
| *AtSAUR16* | At4g38860  (Yes) | In the hypocotyls, cotyledons, rosette leaves, cauline leaves, flower and silique, and be induced by R:FR light | AtSAUR16 promotes cells elongation including etiolated hypocotyls, pistils, filaments, sepals, cauline leaves and siliques. | (Bemer et al., 2017) |
| *AtSAUR41* | At1g16510  (Yes) | In the quiescent center, cortex/endodermis initials, the endodermal layer, stem cell niches of lateral root primordia, root meristems. | AtSAUR41 overexpression lines shows long hypocotyls, increased vegetative biomass and lateral root development, expanded petals and twisted inflorescence stems.  (AtSAUR41 located in the cytoplasm) | (Kong et al., 2013) |
| *AtSAUR62* | At1g29430  (Yes) | In elongating pollen tubes, pollen grains, seedlings, stems, rosette leaves, cauline leaves, flowers, siliques, and be induced by pollination. | The *atsaur62* mutant have many aborted seeds, normal pollen viability but defective in vitro and in vivo pollen tube growth, with branching phenotypes and unfertilized seeds in mature siliques.  (SAUR62 located in the nucleus) | (He et al., 2018) |
| *AtSAUR10* | At2g18010  (Yes) | At the abaxial side of branches. At*SAUR10* can be induced by low R:FR light, auxin, BR, shade, and a combination of auxin and brassinosteroids. | *AtSAUR10* overexpression lines shows longer etiolated hypocotyls, pistils, filaments, sepals, cauline leaves, etiolated hypocotyls, siliques and a wavy stem. | (Bemer et al., 2017; van Mourik et al., 2017) |
| *AtSAUR65* | At1g29460  (Yes) | In hypocotyls, and be induced by auxin. | *AtSAUR65* overexpression lines shows longer hypocotyls, larger cotyledon areas, opened cotyledons. | (Sun et al., 2016) |
| *AtSAUR75* | At5g27780  (Yes) | In elongating pollen tubes, flowers, pollen grains, pollen tubes, and ovules. *AtSAUR75* can be induced by pollination. | Pollen tube growth of *atsaur75* mutants is defective with branching phenotypes, resulting in many aborted seeds, unfertilized seeds in mature siliques.  (AtSAUR75 located in the nucleus) | (He et al., 2018) |
| *AtSAUR26/27/28* | At3g03840/  At3g03830/  At3g03820  (Yes) | In leaves, especially higher in petiole. *AtSAUR27* can be induced by high temperature) | AtSAUR26 subfamily is a key variation for thermo-responsive architecture. | (Wang et al., 2019) |
| *AtSAUR8* | At2g16580  (Yes) | In petiole and midvein of leaves. | *AtSAUR10*-clade genes generally induce cell-elongation such as etiolated hypocotyls. | (van Mourik et al., 2017) |
| *OsSAUR39* | Os09g37330  (Yes) | older leaves and can be induced by auxin, nitrogen, salinity, cytokinin, and anoxia. | *OsSAUR39* overexpression reduced auxin production and transport, resulting in smaller vascular tissue, reduced lateral root development, lower yield, lower shoot and root lengths, and more seedling/rosette growth.  (OsSAUR39 located in the cytoplasm) | (Kant et al., 2009) |
| *OsSAUR45* | Os09g37400  (Yes) | leaf blade and the vascular tissue of the leaf sheaths and stems, and can be induced by 6-BA, IAA, NAA, and 2,4- D, ABA, TIBA, NPA, NOA and CHPAA. | OsSAUR45 is involved in plant growth by affecting auxin synthesis and transport. The overexpression lines displayed pleiotropic developmental defects including reduced plant height and primary root length, fewer adventitious roots, narrower leaves, and reduced seed setting.  (OsSAUR45 located in the cytoplasm) | (Jain et al., 2006) |
| *OsSAUR5* | Os09g37460  (Yes) | Leaf, and can be induced by infection of the pathogen and exogenous auxin treatment. | *OsSAUR51* may play a role in promoting bacterial blight resistance in plants. | (Aoki et al., 2016) |
| *SLSAUR69* | Sl09G008170  (Yes) | Ripening fruit, and is up-regulated at the transcription level by both auxin and ethylene | *SlSAUR69* regulate polar auxin transport, controlling premature initiation of ripening.  (SiSAUR69 located in the nuclear) | (Shin et al., 2019) |
| *TaSAUR75* | Treas_5DL_32B83E57E  (Yes) | *TaSAUR75* is repressed by salt stress in wheat | *TaSAUR75* may increase tolerance to drought and salt stress by upregulating stress-responsive genes under abiotic stress. | (Guo et al., 2018) |
| *AtSAUR19/24* | At5g18010  At5g18080  (Yes) | In elongating tissues. | *AtSAUR19* promote cell expansion which result in increasing leaf size and vegetative biomass.  (AtSAUR19/24 located in plasma membrane) | (Spartz et al., 2012) |
| *AtSAUR14* | At4g38840  (Yes) | In cotyledons during the dark to light transition. | The overexpression of *AtSAUR14* shows longer hypocotyls, opened cotyledons. | (Sun et al., 2016) |
| *AtSAUR78* | At1g72430  (Yes) | In seedling, root, leaf, flowers and silique, and can be induced by auxin, NAA and ethylene. | SAUR78 may affect ethylene receptor signaling and promote plant growth in *Arabidopsis*.  (AtSAUR78 located in cytoplasm, membrane and nucleus) | (Li et al., 2015) |
| *OsSAUR5* | Os02g05060 | roots | *Ossaur5* mutants showed dwarfism, sterility, late heading, low tillering, and altered yield. | (Jain et al., 2006) |
| *OsSAUR7/13/31/52* | Os02g20320/ Os03g18050/ Os03g18050/ Os09g37470 | etiolated seedlings, and can be induced by auxin in etiolated rice coleoptiles. | *OsSAUR7, OsSAUR13, OsSAUR31, OsSAUR52 and OsSAUR18* may interact with light and hormone. |  |
| *OsSAUR18* | Os04g43740 | green seedlings |  |  |
| *AtSAUR71* | At1g56150  (Yes) | In root tip cells, steles of young roots, hypocotyls, guard mother cells, young guard cells of cotyledons and leaves. | (AtSAUR71 located in cytoplasm) | (Qiu et al., 2013) |
| *AtSAUR40* | At1g79130  (Yes) | In root tip cells. | (AtSAUR40 located in cytoplasm) | (Qiu et al., 2013) |
| *OsSAUR54* | Os09g37490 | rice stigma | *OsSAUR54* may promote pollen tube growth. | (Li et al., 2007) |
| *ZmSAUR1* | Zm2g452996 |  | *ZmSAUR1* and *ZmSAUR2* were involved in auxin-mediated cell elongation. | (Yang and Poovaiah, 2000; Chen et al., 2008) |
| *ZmSAUR6/38* | Zm2g059138Zm2g076345 | In seed growth | *ZmSAUR6* and *ZmSAUR38* may be involved in seed development | (Chen et al., 2014) |
| *ZmSAUR43/63* | Zm2g410499Zm2g430052 | in anthers | *ZmSAUR43* and *ZmSAUR63* may play an important role in anthers due to high expression levels. |  |
| *ZmSAUR31* | Zm2g156451 | In coleoptile primary root | It might be involved in the growth and development of coleoptile primary root in maize. |  |
| *PheSAUR5/31/36* | Ph01000057G0880/ Ph01003710G0040/ Ph01006019G0010 | They were up-regulated in the leaves after IAA treatment. | They are involved in the developments of leaf in seedlings | (Bai et al., 2017) |
| *CitSAUR65* | Orange1.1g046189m.g b | *CitSAUR65* was slightly increased first and then decreased to a low-level IAA. | They are effective negative regulator in IAA-induced delay in abscission.  (CitSAUR65 located in mitochondrial and nuclear) | (Xie et al., 2015) |
| *PheSAUR2* | Ph01000003G0680 | Stem | They may play important roles in promoting shoot elongation  (PheSAUR2 located in nuclear) | (Bai et al., 2017) |
| *PheSAUR15* | Ph01000407G0060 | Stem and can be induced by IAA in the leaves. | They may be involved in cell division in shoot growth and the developments of leaf seedlings.  (PheSAUR15 located in nuclear) |  |
| *PheSAUR20* | Ph01001832G0020 | Stem and roots. | They may be involved in cell division in shoot growth.  (PheSAUR20 located in mitochondrial) |  |
| *AbSAUR1* | (Yes) | Roots | *AbSAUR1* overexpression showed increased plant height, thickened stems, increased number of branches, leaves and roots. | (Wei et al., 2006; Bai et al., 2019) |
| *AtSAUR4930/39/41/72* | At4g34750  At4g34750At5g53590 At3g43120At1g16510 At3g12830  (yes) | In senescent leaves | AtSAUR49/30/39/41/72 can positively regulate leaf senescence by suppressing SSPP in *Arabidopsis.*  (AtSAUR49/30/39/41/72 located in cytoplasm) | （Zewen Wen et al. 2019） |
| *AtSAUR* *40/41/71/*  *72* | At1g79130/ At1g16510/ At1g56150/ At3g12830  (Yes) | In young seedlings | The SAUR41 subfamily is abscisic acid inducible to modulate cell expansion and salt tolerance. | (Ting Qiu et al. 2019) |

References

Aoki, H., Onishi, A., Miyashita, M., Miyagawa, H., Yatou, O. and Saito, K., 2016. Involvement of the rice *OsSAUR51* gene in the auxin-related field resistance mechanism against bacterial blight disease. Jarq-Japan Agricultural Research Quarterly 50, 219-227.

Bai, F., Li, S., Yang, C., Zhao, T., Zhang, T., Lan, X., Chen, M. and Liao, Z., 2019. Overexpression of the *AbSAUR1* gene enhanced biomass production and alkaloid yield in *Atropa belladonna*. Industrial Crops and Products 140, 111705.

Bai, Q., Hou, D., Li, L., Cheng, Z., Ge, W., Liu, J., Li, X., Mu, S. and Gao, J., 2017. Genome-wide analysis and expression characteristics of small auxin-up RNA (*SAUR*) genes in moso bamboo (*Phyllostachys edulis*). Genome 60, 325-336.

Bemer, M., van Mourik, H., Muino, J.M., Ferrandiz, C., Kaufmann, K. and Angenent, G.C., 2017. FRUITFULL controls *SAUR10* expression and regulates *Arabidopsis* growth and architecture. J Exp Bot 68, 3391-3403.

Chae, K., Isaacs, C.G., Reeves, P.H., Maloney, G.S., Muday, G.K., Nagpal, P. and Reed, J.W., 2012. *Arabidopsis* SMALL AUXIN UP RNA63 promotes hypocotyl and stamen filament elongation. Plant J 71, 684-97.

Chen, J.Q., Meng, X.P., Zhang, Y., Xia, M. and Wang, X.P., 2008. Over-expression of *OsDREB* genes lead to enhanced drought tolerance in rice. Biotechnol Lett 30, 2191-8.

Chen, Y., Hao, X. and Cao, J., 2014. Small auxin upregulated RNA (*SAUR*) gene family in maize: identification, evolution, and its phylogenetic comparison with *Arabidopsis*, rice, and sorghum. J Integr Plant Biol 56, 133-50.

Guo, Y., Jiang, Q., Hu, Z., Sun, X., Fan, S. and Zhang, H., 2018. Function of the auxin-responsive gene *TaSAUR75* under salt and drought stress. The Crop Journal 6, 181-190.

He, S.L., Hsieh, H.L. and Jauh, G.Y., 2018. SMALL AUXIN UP RNA62/75 are required for the translation of transcripts essential for pollen tube growth. Plant Physiol 178, 626-640.

Hou, K., Wu, W. and Gan, S.S., 2013. *SAUR36*, a small auxin up RNA gene, is involved in the promotion of leaf senescence in *Arabidopsis*. Plant Physiol 161, 1002-9.

Jain, M., Tyagi, A.K. and Khurana, J.P., 2006. Genome-wide analysis, evolutionary expansion, and expression of early auxin-responsive SAUR gene family in rice (*Oryza sativa*). Genomics 88, 360-71.

Kant, S., Bi, Y.M., Zhu, T. and Rothstein, S.J., 2009. *SAUR39*, a small auxin-up RNA gene, acts as a negative regulator of auxin synthesis and transport in rice. Plant Physiol 151, 691-701.

Kong, Y., Zhu, Y., Gao, C., She, W., Lin, W., Chen, Y., Han, N., Bian, H., Zhu, M. and Wang, J., 2013. Tissue-specific expression of SMALL AUXIN UP RNA41 differentially regulates cell expansion and root meristem patterning in *Arabidopsis*. Plant Cell Physiol 54, 609-21.

Li, M., Xu, W., Yang, W., Kong, Z. and Xue, Y., 2007. Genome-wide gene expression profiling reveals conserved and novel molecular functions of the stigma in rice. Plant Physiol 144, 1797-812.

Li, Z.G., Chen, H.W., Li, Q.T., Tao, J.J., Bian, X.H., Ma, B., Zhang, W.K., Chen, S.Y. and Zhang, J.S., 2015. Three SAUR proteins SAUR76, SAUR77 and SAUR78 promote plant growth in *Arabidopsis*. Sci Rep 5, 12477.

Markakis, M.N., Boron, A.K., Van Loock, B., Saini, K., Cirera, S., Verbelen, J.P. and Vissenberg, K., 2013. Characterization of a small auxin-up RNA (*SAUR*)-like gene involved in *Arabidopsis thaliana* development. PLoS One 8, e82596.

Park, J.-E., Kim, Y.-S., Yoon, H.-K. and Park, C.-M., 2007. Functional characterization of a small auxin-up RNA gene in apical hook development in *Arabidopsis*. Plant Science 172, 150-157.

Qiu, T., Chen, Y., Li, M., Kong, Y., Zhu, Y., Han, N., Bian, H., Zhu, M. and Wang, J., 2013. The tissue-specific and developmentally regulated expression patterns of the *SAUR41* subfamily of small auxin up RNA genes: potential implications. Plant Signal Behav 8.

Shin, J.H., Mila, I., Liu, M., Rodrigues, M.A., Vernoux, T., Pirrello, J. and Bouzayen, M., 2019. The RIN-regulated Small Auxin-Up RNA SAUR69 is involved in the unripe-to-ripe phase transition of tomato fruit via enhancement of the sensitivity to ethylene. New Phytol 222, 820-836.

Spartz, A.K., Lee, S.H., Wenger, J.P., Gonzalez, N., Itoh, H., Inze, D., Peer, W.A., Murphy, A.S., Overvoorde, P.J. and Gray, W.M., 2012. The *SAUR19* subfamily of SMALL AUXIN UP RNA genes promote cell expansion. Plant J 70, 978-90.

Spartz, A.K., Ren, H., Park, M.Y., Grandt, K.N., Lee, S.H., Murphy, A.S., Sussman, M.R., Overvoorde, P.J. and Gray, W.M., 2014. SAUR inhibition of PP2C-D phosphatases activates plasma membrane H^+^-ATPases to promote cell expansion in *Arabidopsis*. Plant Cell 26, 2129-2142.

Stamm, P. and Kumar, P.P., 2013. Auxin and gibberellin responsive *Arabidopsis* SMALL AUXIN UP RNA36 regulates hypocotyl elongation in the light. Plant Cell Rep 32, 759-69.

Sun, N., Wang, J., Gao, Z., Dong, J., He, H., Terzaghi, W., Wei, N., Deng, X.W. and Chen, H., 2016. Arabidopsis SAURs are critical for differential light regulation of the development of various organs. Proc Natl Acad Sci U S A 113, 6071-6.

van Mourik, H., van Dijk, A.D.J., Stortenbeker, N., Angenent, G.C. and Bemer, M., 2017. Divergent regulation of *Arabidopsis* SAUR genes: a focus on the SAUR10-clade. BMC Plant Biol 17, 245.

Wang, Z., Yang, L., Liu, Z., Lu, M., Wang, M., Sun, Q., Lan, Y., Shi, T., Wu, D. and Hua, J., 2019. Natural variations of growth thermo-responsiveness determined by SAUR26/27/28 proteins in *Arabidopsis thaliana*. New Phytol 224, 291-305.

Wei, H.B., Cui, B.M., Ren, Y.L., Li, J.H., Liao, W.B., Xu, N.F. and Peng, M., 2006. Research progresses on auxin response factors. Journal of Integrative Plant Biology 48, 622-627.

Xie, R., Dong, C., Ma, Y., Deng, L., He, S., Yi, S., Lv, Q. and Zheng, Y., 2015. Comprehensive analysis of SAUR gene family in *citrus* and its transcriptional correlation with fruitlet drop from abscission zone A. Funct Integr Genomics 15, 729-40.

Yang, T. and Poovaiah, B.W., 2000. Molecular and biochemical evidence for the involvement of calcium/calmodulin in auxin action. The Journal of Biological Chemistry 275, 3137-43.
