# Supplementary material for: Genome-wide identification and characterization of small auxin-up RNA (SAUR) gene family in plants: evolution and expression profiles during normal growth and stress response
Source: BMC Plant Biol. 2021 Jan 6;21:4. doi: 10.1186/s12870-020-02781-x (PMC7789510; doi:10.1186/s12870-020-02781-x)
Supplement: Supplementary file 7 — Additional file 7: Supplementary Fig. 1. Maximum likelihood phylogenetic tree constructed by IQ-TREE of the SAUR gene family from the thirteen plant species. [file 12870_2020_2781_MOESM7_ESM.docx]

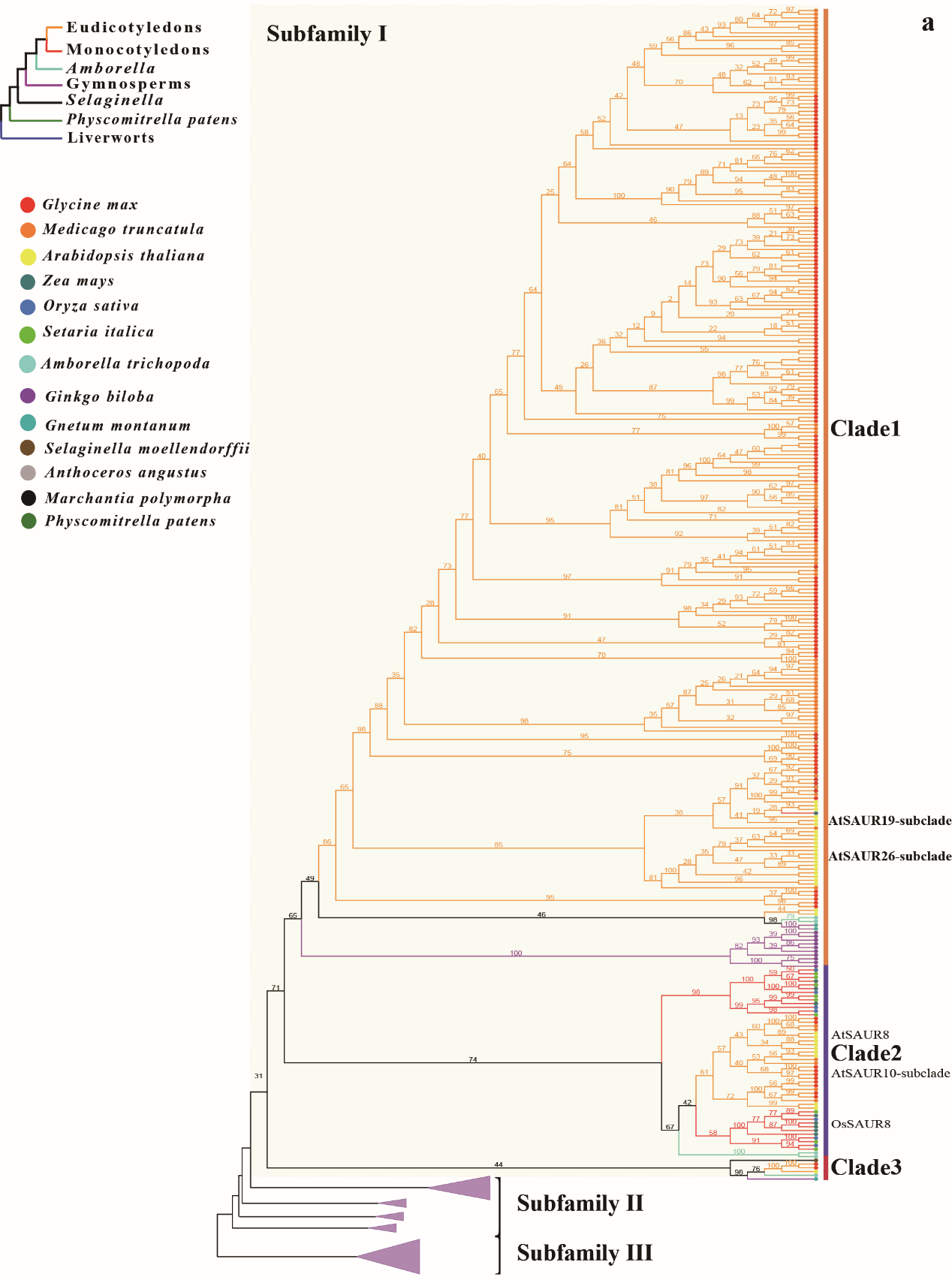


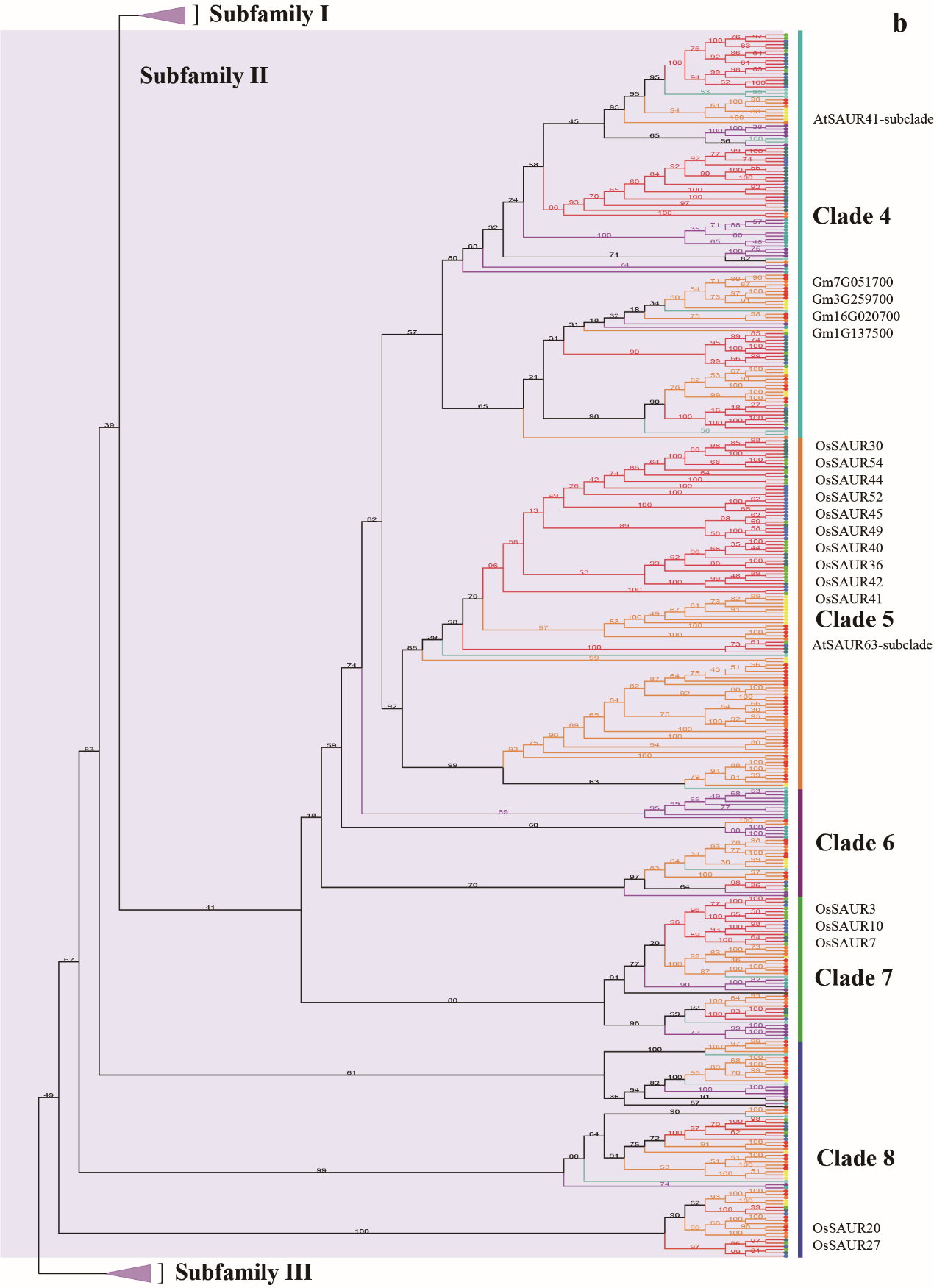


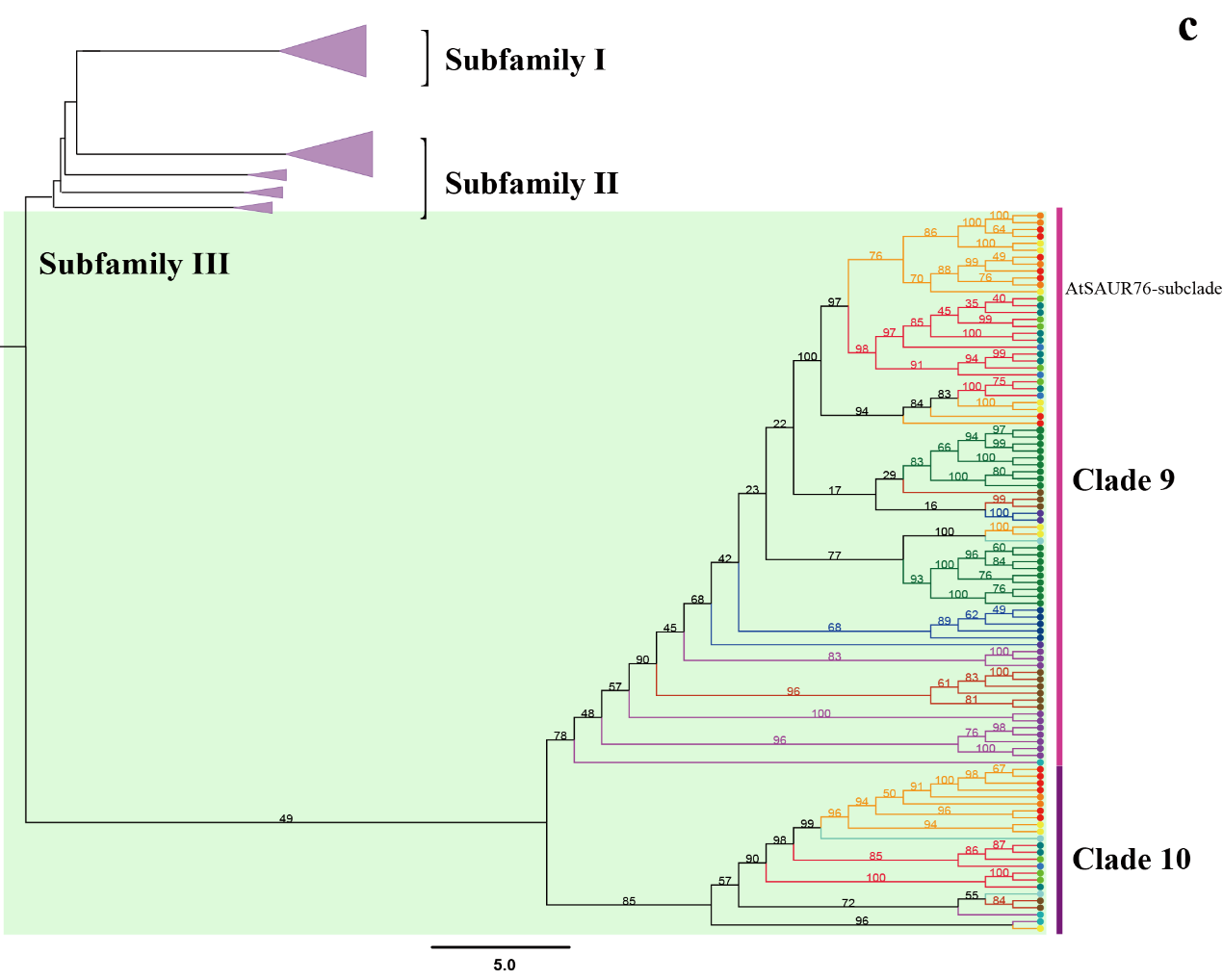


Supplementary Figure 1. Maximum likelihood phylogenetic tree constructed by IQ-TREE of the SAUR gene family.

The ML tree was constructed from multiple sequences alignment of all SAUR proteins from *Anthoceros angustus, Marchantia polymorpha*, *Physcomitrella patens, Selaginella moellendorffii*, *Ginkgo biloba*, *Gnetum montanum*, *Amborella trichopoda,*  *Arabidopsis thaliana*, *Oryza sativa*, *Zea mays*, *Setaria italica*, *Glycine max* and *Medicago truncatula* using IQ-TREE v2.0.6 with JTT+R9 model. Then SAUR members were clarified in FigTree v1.4.4. There SAUR family had been divided into three subfamily and 10 clades. Bootstrap support rates were labeled at corresponding branches. SAUR subclades reported and members mentioned in the article were also labeled on it. Branches leading to genes from the different phyla were colored according to the simplified phylogeny of land plants that is shown in the top left corner. Different colorful dots were used to represent SAUR members from different species.
