# Supplementary material for: Genome-wide identification and characterization of small auxin-up RNA (SAUR) gene family in plants: evolution and expression profiles during normal growth and stress response
Source: BMC Plant Biol. 2021 Jan 6;21:4. doi: 10.1186/s12870-020-02781-x (PMC7789510; doi:10.1186/s12870-020-02781-x)
Supplement: Supplementary file 8 — Additional file 8: Supplementary Fig. 2. Maximum likelihood phylogenetic tree constructed by FastTree of the SAUR gene family in Arabidopsis thaliana, Mimosa pudica, Cercis canadensis, Medicago truncatula and Glycine max. [file 12870_2020_2781_MOESM8_ESM.docx]

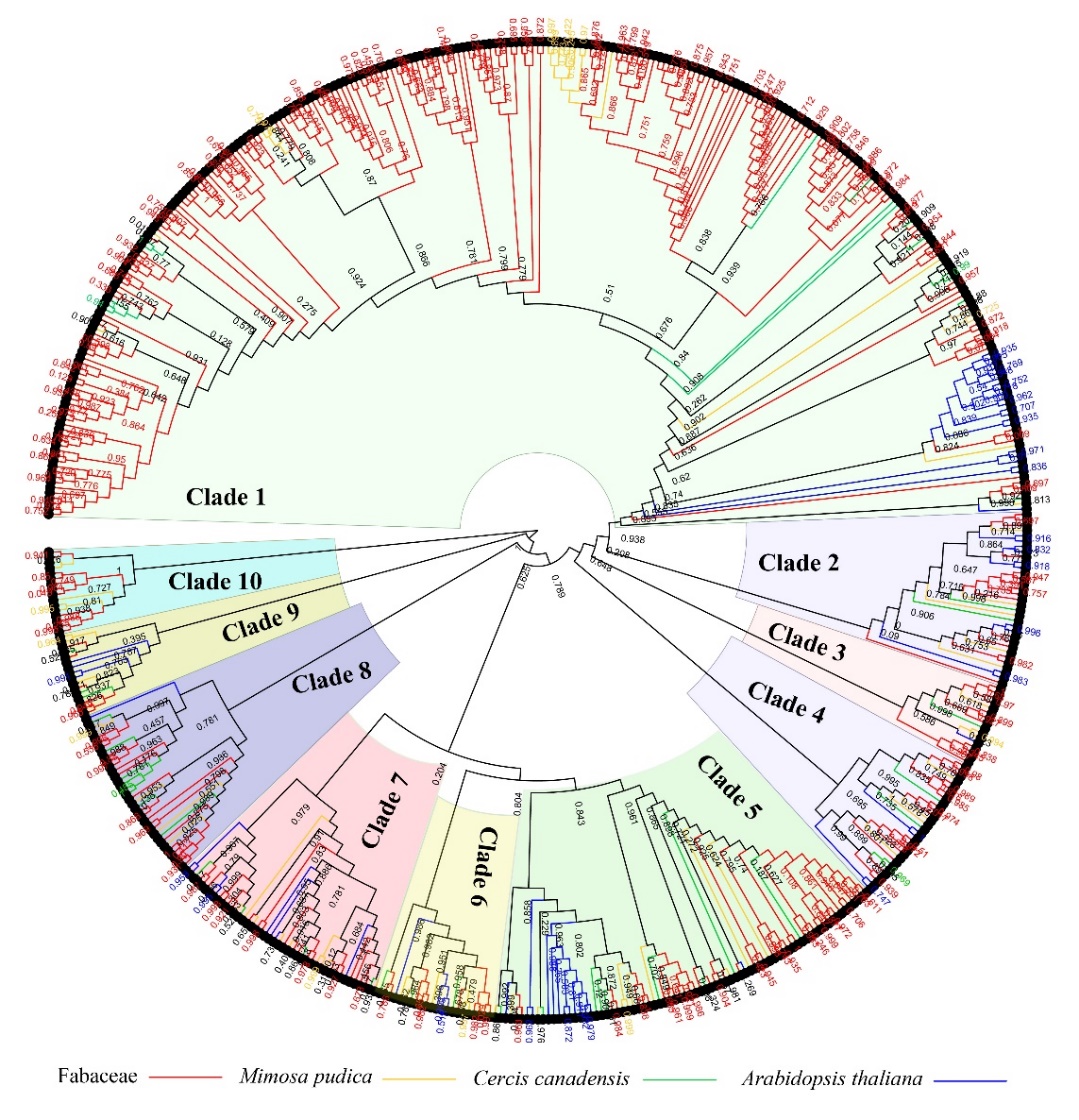


Supplementary Figure 2. Maximum likelihood phylogenetic tree constructed by FastTree of the *SAUR* gene family in *Arabidopsis thaliana*, *Mimosa pudica*, *Cercis canadensis*, *Medicago truncatula* and *Glycine max*.

The ML tree was constructed using FastTree v2.1 with the JTT+CAT model. Then SAUR members were clarified in FigTree v1.4.4. The *SAUR* family had been divided into 10 clades. Bootstrap support rates were labeled at corresponding branches. Different colorful branches were used to represent SAUR members from different species.
