# Supplementary material for: Genome-wide identification and characterization of small auxin-up RNA (SAUR) gene family in plants: evolution and expression profiles during normal growth and stress response
Source: BMC Plant Biol. 2021 Jan 6;21:4. doi: 10.1186/s12870-020-02781-x (PMC7789510; doi:10.1186/s12870-020-02781-x)
Supplement: Supplementary file 9 — Additional file 9: Supplementary Fig. 3. Gene synteny analysis between Fabaceae plants and Arabidopsis thaliana. [file 12870_2020_2781_MOESM9_ESM.docx]

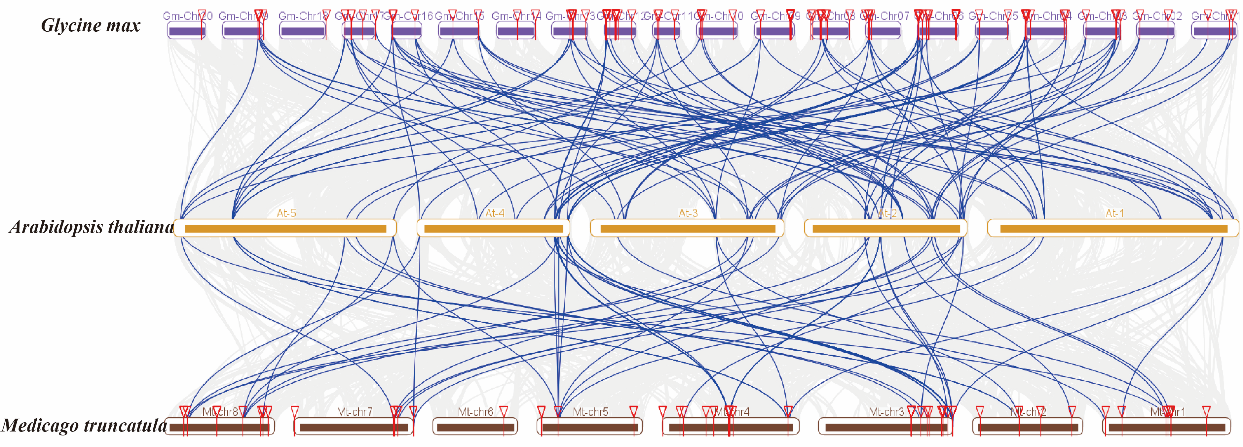


Supplementary Figure 3. Gene synteny analysis between Fabaceae plants and *Arabidopsis thaliana*.

The genes synteny was built with MCScanX program in TBtools. *Glycine max* and *Medicago truncatula* genes were linked to their homologous genes in *Arabidopsis thaliana*, respectively. Synteny relationship of *SAUR* genes was highlighted with blue lines.
