# Supplementary material for: Genome-wide identification and characterization of small auxin-up RNA (SAUR) gene family in plants: evolution and expression profiles during normal growth and stress response
Source: BMC Plant Biol. 2021 Jan 6;21:4. doi: 10.1186/s12870-020-02781-x (PMC7789510; doi:10.1186/s12870-020-02781-x)
Supplement: Supplementary file 10 — Additional file 10: Supplementary Fig. 4. The five conserved motifs of SAUR proteins from seven plant species containing Arabidopsis thaliana, Oryza sativa, Zea mays, Glycine max, Medicago truncatula, Setaria italica, Physcomitrella patens. [file 12870_2020_2781_MOESM10_ESM.docx]

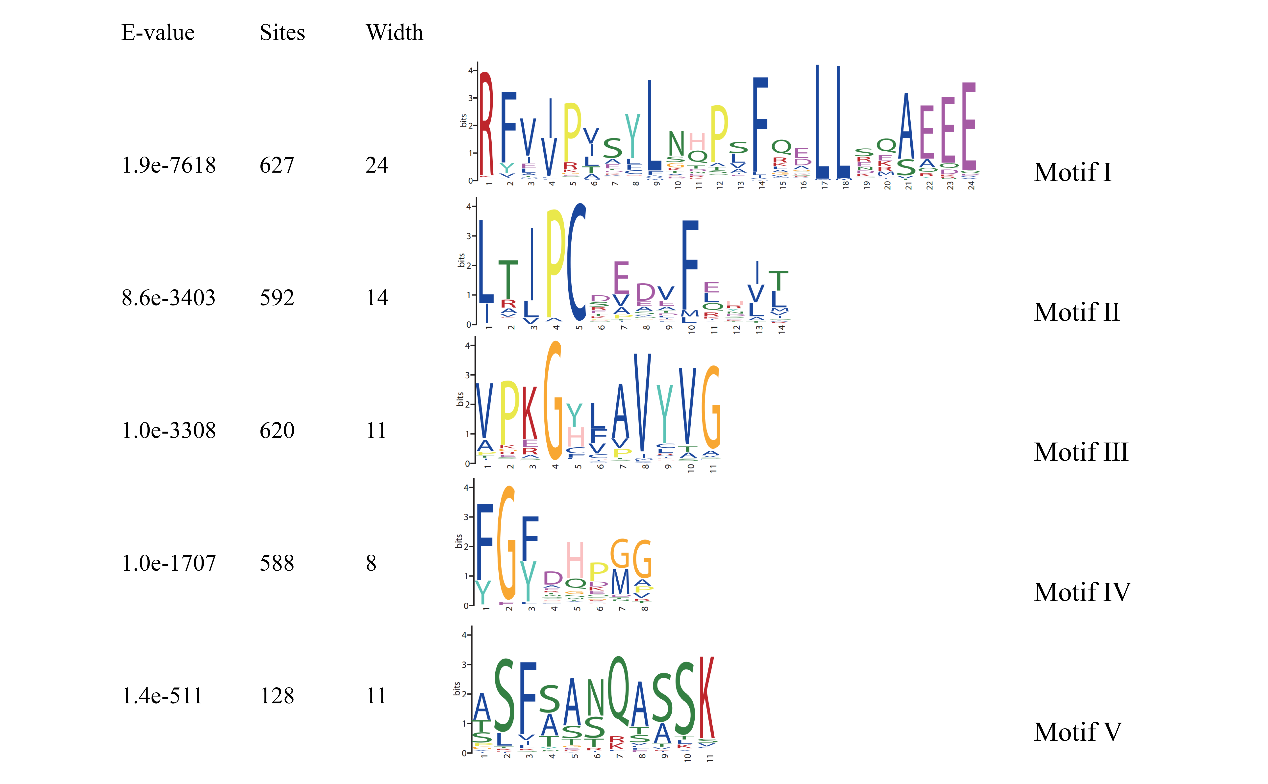


Supplementary Figure 4. The five conserved consensus motifs of SAUR proteins in the seven plant species containing *Arabidopsis thaliana*, *Oryza sativa*, *Zea mays*, *Glycine max*, *Medicago truncatula*, *Setaria italica* and *Physcomitrella patens*. The symbol heights represent the relative frequency of each residue. The numbers of sites, width and e-value for each motif are also shown.
