# Supplementary material for: Genome-wide identification and characterization of small auxin-up RNA (SAUR) gene family in plants: evolution and expression profiles during normal growth and stress response
Source: BMC Plant Biol. 2021 Jan 6;21:4. doi: 10.1186/s12870-020-02781-x (PMC7789510; doi:10.1186/s12870-020-02781-x)
Supplement: Supplementary file 11 — Additional file 11: Supplementary Fig. 5. Expression profiles of small auxin-up RNA (SAUR) genes in soybean (Glycine max). [file 12870_2020_2781_MOESM11_ESM.docx]

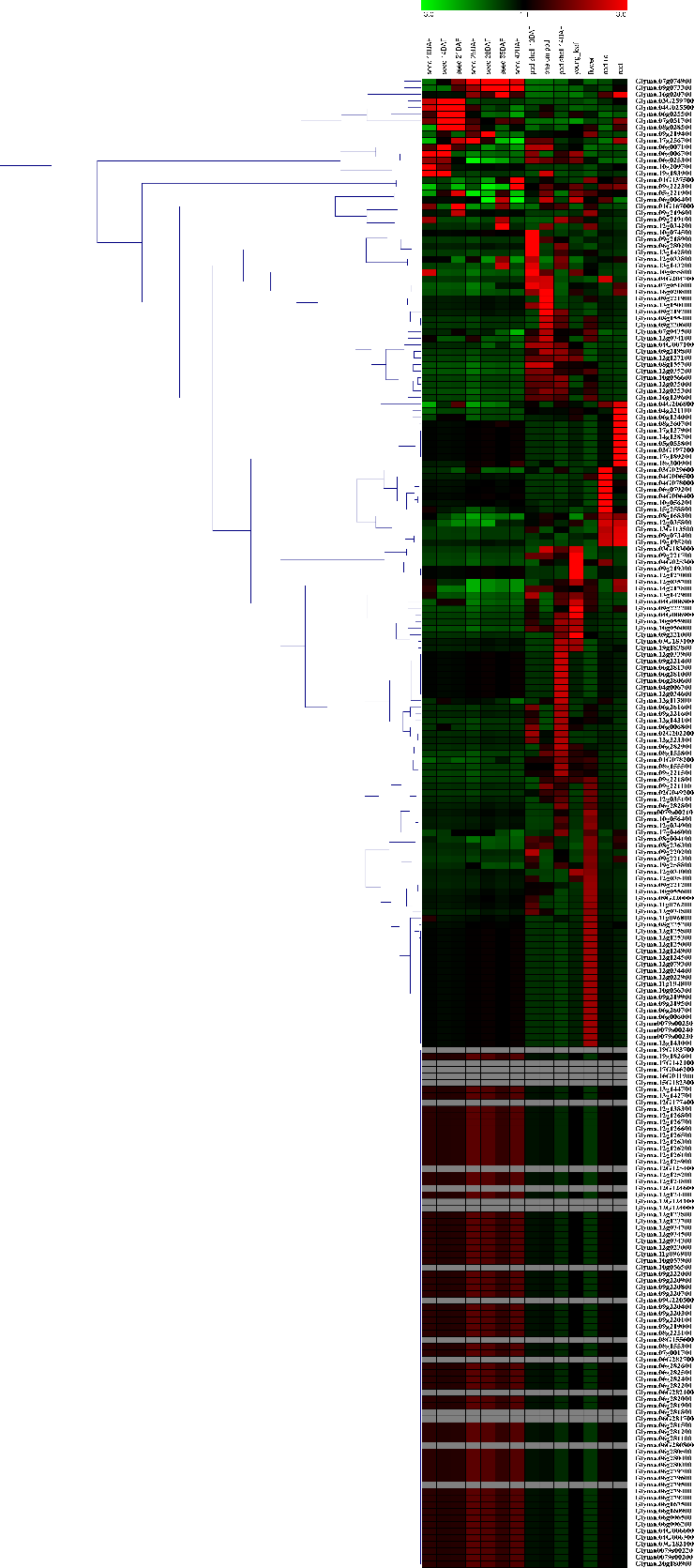


Supplementary Figure 5. Expression profiles of small auxin‐up RNA (*SAUR*) genes in soybean. *GmSAURs* gene expression values of 17 different development tissues or organ systems by RNA-seq. DAF: Days After Flowers.
