# Supplementary material for: Genome-wide identification and characterization of small auxin-up RNA (SAUR) gene family in plants: evolution and expression profiles during normal growth and stress response
Source: BMC Plant Biol. 2021 Jan 6;21:4. doi: 10.1186/s12870-020-02781-x (PMC7789510; doi:10.1186/s12870-020-02781-x)
Supplement: Supplementary file 12 — Additional file 12: Supplementary Fig. 6. Digital gene expression of OsSAURs from different developmental stage of rice varieties under diverse stress conditions. [file 12870_2020_2781_MOESM12_ESM.docx]

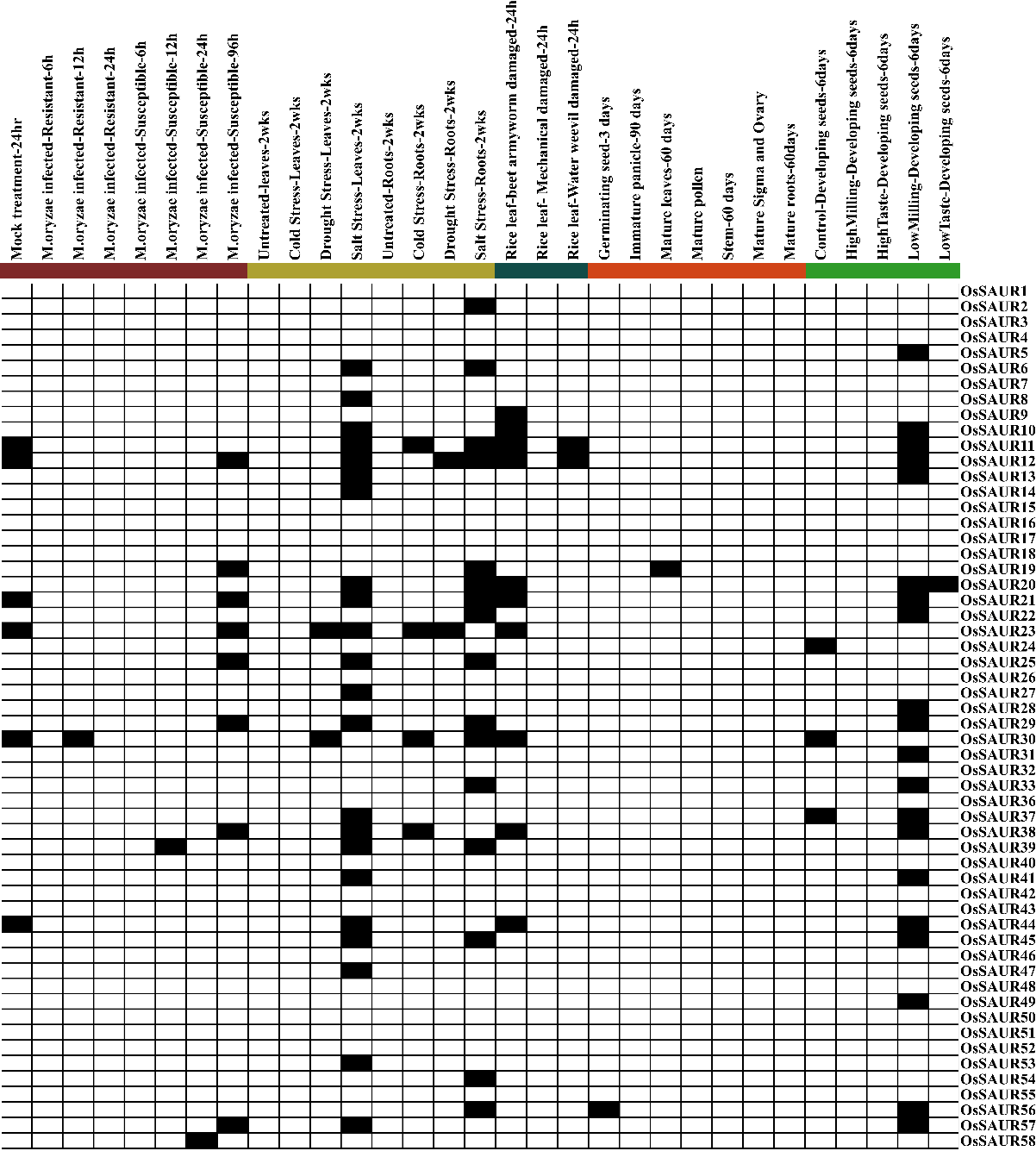


Supplementary Figure 6. Digital gene expression of *OsSAURs* under diverse stress, in different developmental stage and from different rice varieties. Based on digital gene expression (DGE) libraries, genes were regarded as “expressed” if at least one sequence read mapped uniquely within an exon. Black represents “expressed” while white means “noexpressed”.
