# Supplementary material for: Genome-wide identification and characterization of small auxin-up RNA (SAUR) gene family in plants: evolution and expression profiles during normal growth and stress response
Source: BMC Plant Biol. 2021 Jan 6;21:4. doi: 10.1186/s12870-020-02781-x (PMC7789510; doi:10.1186/s12870-020-02781-x)
Supplement: Supplementary file 13 — Additional file 13: Supplementary Fig. 7. The number of identified SAUR genes from genomes annotated monocotyledons and dicotyledons. [file 12870_2020_2781_MOESM13_ESM.docx]

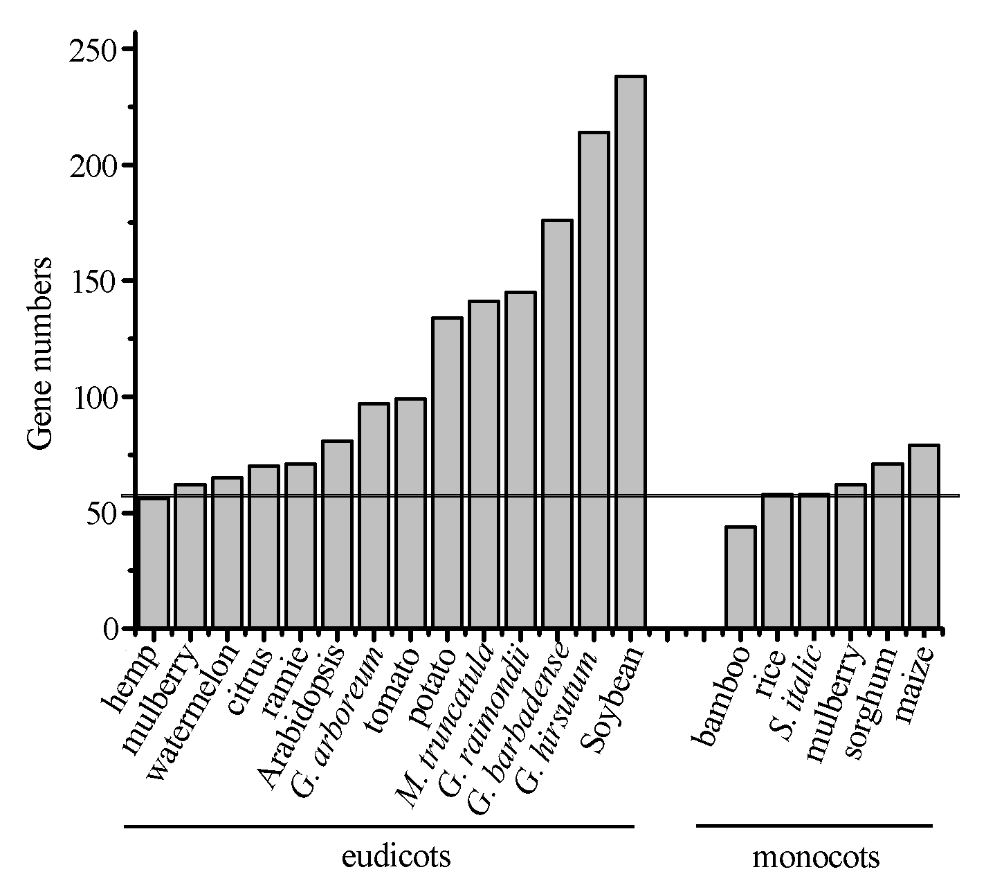


Supplementary Figure 7. The number of identified SAUR genes from genome annotated monocotyledons and dicotyledons. These contains our results and others. The reference list is:

Bai, Q., Hou, D., Li, L., Cheng, Z., Ge, W., Liu, J., Li, X., Mu, S. and Gao, J., 2017. Genome-wide analysis and expression characteristics of small auxin-up RNA (*SAUR*) genes in moso bamboo (*Phyllostachys edulis*). Genome 60, 325-336.

Huang, X., Bao, Y.N., Wang, B., Liu, L.J., Chen, J., Dai, L.J., Baloch, S.U. and Peng, D.X., 2016. Identification of small auxin-up RNA (*SAUR*) genes in *Urticales* plants: mulberry (*Morus notabilis*), hemp (*Cannabis sativa*) and ramie (*Boehmeria nivea*). Journal of Genetics 95, 119-129.

Li, X., Liu, G., Geng, Y., Wu, M., Pei, W., Zhai, H., Zang, X., Li, X., Zhang, J., Yu, S. and Yu, J., 2017. A genome-wide analysis of the small auxin-up RNA (*SAUR*) gene family in cotton. BMC Genomics 18, 815.

Nakano, T., Suzuki, K., Fujimura, T. and Shinshi, H., 2006. Genome-wide analysis of the ERF gene family in *Arabidopsis* and rice. Plant Physiol 140, 411-32.

Xie, R., Dong, C., Ma, Y., Deng, L., He, S., Yi, S., Lv, Q. and Zheng, Y., 2015. Comprehensive analysis of SAUR gene family in citrus and its transcriptional correlation with fruitlet drop from abscission zone A. Funct Integr Genomics 15, 729-40.

Zhao, Y., Chang, X., Qi, D., Dong, L., Wang, G., Fan, S., Jiang, L., Cheng, Q., Chen, X., Han, D., Xu, P. and Zhang, S., 2017. A novel soybean ERF transcription factor, *GmERF113*, increases resistance to *phytophthora sojae* infection in soybean. Front Plant Sci 8, 299.
