# Supplementary material for: Genome-wide identification and characterization of small auxin-up RNA (SAUR) gene family in plants: evolution and expression profiles during normal growth and stress response
Source: BMC Plant Biol. 2021 Jan 6;21:4. doi: 10.1186/s12870-020-02781-x (PMC7789510; doi:10.1186/s12870-020-02781-x)
Supplement: Supplementary file 14 — Additional file 14: Supplementary Fig. 8. Analysis of structural parameter RMSD and Ramachandran plot for structure optimization. [file 12870_2020_2781_MOESM14_ESM.docx]

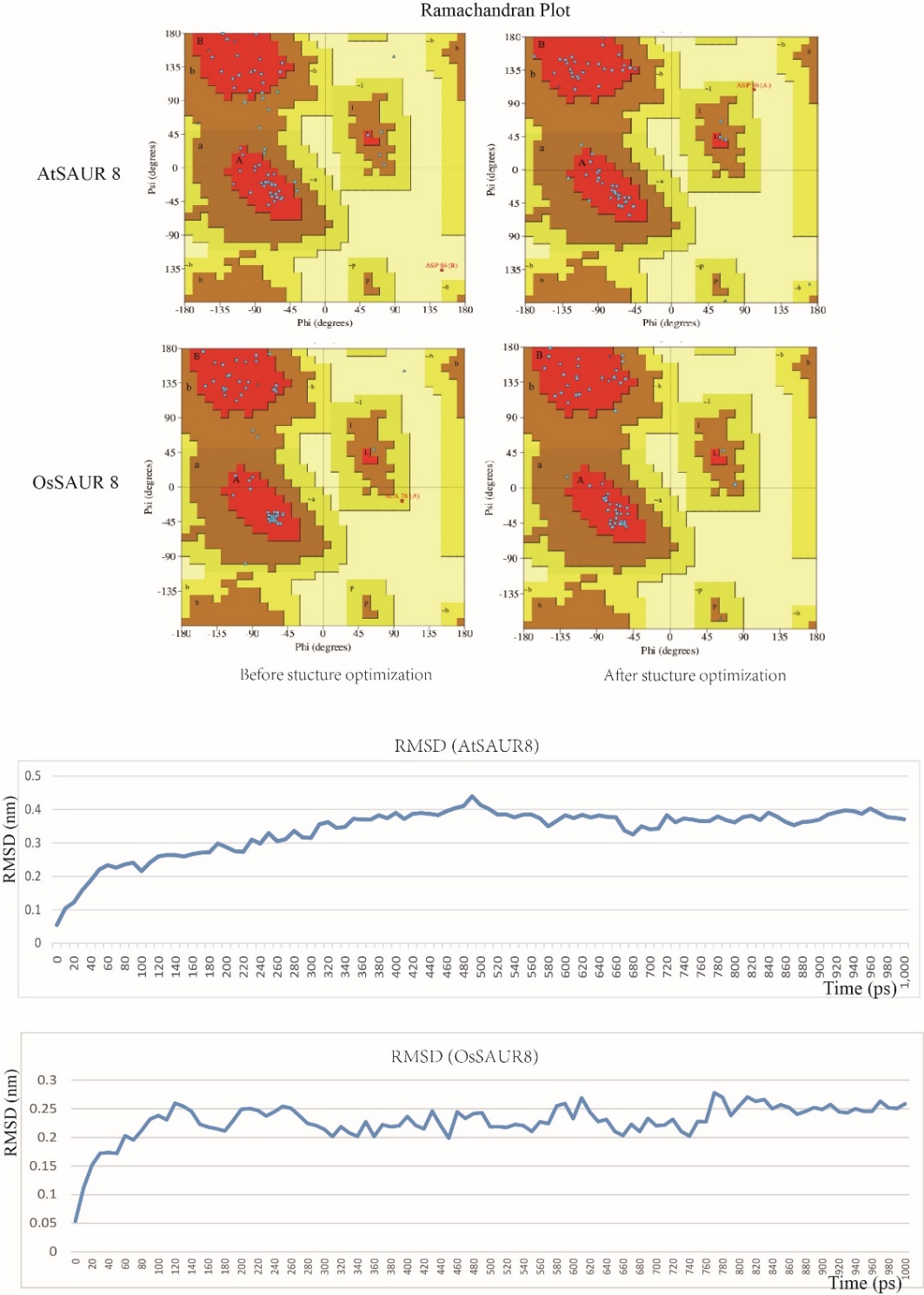


Supplementary Figure 8. Analysis of structural parameter RMSD and Ramachandran plot for structure optimization.

The predicted structures of AtSAUR8 and OsSAUR8 were subjected to MD simulation using GROMACS 2019.1 software package with the Charmm 27 force field and TIPS3P water model in a box large enough to enclose the whole protein. The structure was then subjected to energy minimization with 50000 steps of steepest descent. The minimized structure was equilibrated with NVT and NPT simulation in turn. Finally, we carried out 1 ns long NVT MD simulation and structural parameters like RMSD converged after 800ps.
